# Who Ate Whom? Adaptive *Helicobacter* Genomic Changes That Accompanied a Host Jump from Early Humans to Large Felines

**DOI:** 10.1371/journal.pgen.0020120

**Published:** 2006-07-28

**Authors:** Mark Eppinger, Claudia Baar, Bodo Linz, Günter Raddatz, Christa Lanz, Heike Keller, Giovanna Morelli, Helga Gressmann, Mark Achtman, Stephan C Schuster

**Affiliations:** 1 Department of Biochemistry and Molecular Biology, Center for Comparative Genomics and Bioinformatics, Pennsylvania State University, University Park, Pennsylvania, United States of America; 2 Department of Molecular Biology, Max Planck Institute for Infection Biology, Berlin, Germany; 3 Genomics Group, Max Planck Institute for Developmental Biology, Tübingen, Germany; 4 Center for Infectious Disease Dynamics, Pennsylvania State University, University Park, Pennsylvania, United States of America; The United States Department of Energy Joint Genome Institute, United States of America

## Abstract

Helicobacter pylori infection of humans is so old that its population genetic structure reflects that of ancient human migrations. A closely related species, *Helicobacter acinonychis,* is specific for large felines, including cheetahs, lions, and tigers*,* whereas hosts more closely related to humans harbor more distantly related *Helicobacter* species. This observation suggests a jump between host species. But who ate whom and when did it happen? In order to resolve this question, we determined the genomic sequence of H. acinonychis strain Sheeba and compared it to genomes from H. pylori. The conserved core genes between the genomes are so similar that the host jump probably occurred within the last 200,000 (range 50,000–400,000) years. However, the Sheeba genome also possesses unique features that indicate the direction of the host jump, namely from early humans to cats. Sheeba possesses an unusually large number of highly fragmented genes, many encoding outer membrane proteins, which may have been destroyed in order to bypass deleterious responses from the feline host immune system. In addition, the few Sheeba-specific genes that were found include a cluster of genes encoding sialylation of the bacterial cell surface carbohydrates, which were imported by horizontal genetic exchange and might also help to evade host immune defenses. These results provide a genomic basis for elucidating molecular events that allow bacteria to adapt to novel animal hosts.

## Synopsis

Little is known about the mechanisms that allow jumps of parasites from one host species to a second. We describe the genomic sequence of strain Sheeba of *Helicobacter acinonychis,* a bacterial parasite that can cause severe gastritis of tigers, lions, and cheetahs. The genomic sequence possesses an unusually high number of defective genes that have been fragmented through frameshift mutations, stop codons, and small insertions and deletions. Together with phylogenetic analyses, these observations indicate that H. acinonychis arose 200,000 years ago via a host jump of Helicobacter pylori from early humans. H. acinonychis also possesses five genes involved in sialylation of cell surface carbohydrates that were probably acquired by horizontal gene transfer from unrelated bacteria. Epitopes on the bacterial cell surface were diminished by gene fragmentation (inactivation of outer membrane proteins) and sialylation (masking of carbohydrates), which should facilitate the evasion of feline immune defenses, and may have been crucial for the host jump.

## Introduction

The stomachs of half of the global human population are colonized by the Gram-negative bacterium *Helicobacter pylori,* which results in chronic gastritis and can cause peptic ulcers and gastric cancer [1]. Since the original description of H. pylori in 1984 [2], various *Helicobacter* species have been identified in a wide range of vertebrate hosts, possibly reflecting long-term co-evolution of microbe and host [3]. However, the length of association between H. pylori and humans is controversial and was even suggested to reflect a recent host jump from domesticated animals [4,5]. Domestication occurred during the last 10,000 years, the Neolithic period, and this interpretation would imply that H. pylori has only been associated with humans for less than 10,000 years. Instead, recent data show that H. pylori with East Asian genotypes can be isolated from Amerinds in North and South America [6–8], and thus presumably accompanied human migrations across the Bering Strait, prior to the Neolithic period. Furthermore, the population genetics of H. pylori mimics that of humans, and seems to reflect ancient human migrations [8]. Therefore, humans probably acquired H. pylori quite early in their history, long before the migrations of modern humans out of Africa [9].

**Figure 1:**
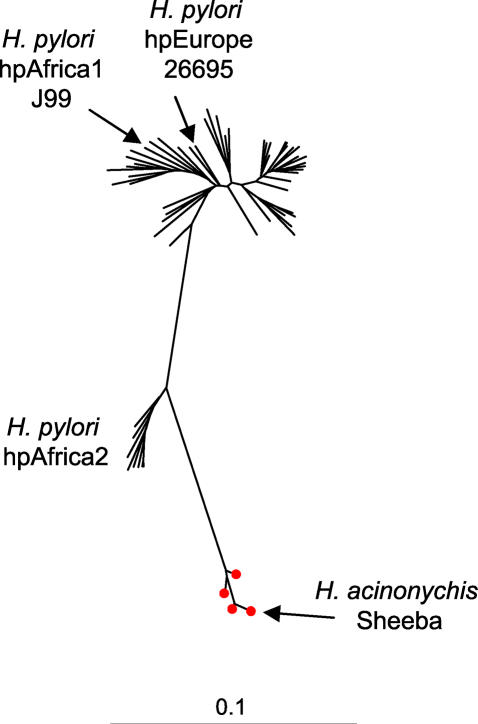
Neighbor-Joining Tree Based on the GTR+G+I Evolutionary Model for 3,406 bp Sequences from 58 Strains of H. pylori and Four Strains of H. acinonychis The tree shows the phylogenetic relationships between H. acinonychis and populations within *H. pylori,* and arrows indicate the three strains, J99, 26695, and Sheeba, from which genome sequences are currently available. This phylogenetic tree indicates that H. pylori (lines) and H. acinonychis (lines plus red dots) are closely related but cannot resolve the direction of ancestor-descendent relationships. Genetic distance scale bar at bottom.

Other than humans, the only natural hosts for H. pylori seem to be non-human primates [10], but *H. pylori* can infect animals such as mice, dogs, and gerbils in laboratory experiments. The stomachs of carnivores (dogs, cats, and cheetahs) are also frequently naturally colonized with *Helicobacter,* but these belong to *Helicobacter felis, Helicobacter bizzozeronii,* and other species that are quite different from H. pylori [3,11,12]. Unusually, one *Helicobacter* species, *Helicobacter acinonychis,* that is closely related to H. pylori according to the sequences of ribosomal RNA and multiple other genes [5,13–15] (Figure 1), colonizes the stomachs of large felines, including cheetahs, tigers, and lions. The existence of H. acinonychis led to the suggestion that H. pylori resulted from a host jump to humans from a non-domesticated zoonotic source [5], such as large felines. However, neutral sequence variation alone cannot indicate the direction of a host jump, which leaves the question of the origins of H. acinonychis unresolved, namely, “who ate whom?” We have therefore determined the genome sequence of H. acinonychis strain Sheeba (Figure S1) and performed comparative genomic analyses with the genomes of H. pylori strains 26695 [16] and J99 [17], as well as other Campylobacterales, to identify signatures of adaptation that might have accompanied a recent host jump.

## Results

### Relatedness between H. acinonychis and H. pylori

**Figure 2:**
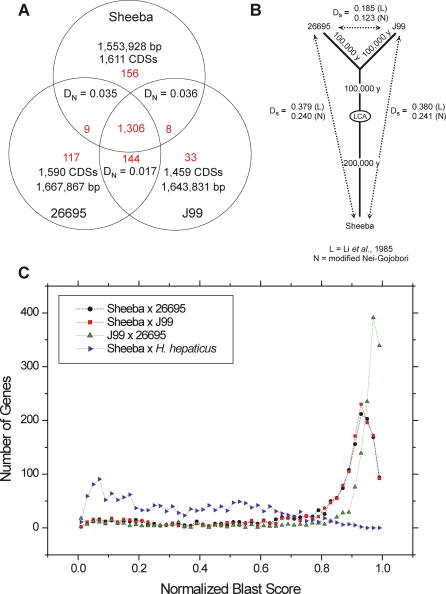
Similarities between the Genomes of H. acinonychis Sheeba and H. pylori 26695 and J99 (A) Venn diagram of genomic properties. Numbers in red within each arc represent numbers of genes, each of which may contain multiple CDSs if the corresponding gene is fragmented. (B) Age calculations since a common ancestor (LCA) based on synonymous pair-wise distances for 612 conserved genes according to the methods of Li et al., 1985 and the modified Nei-Gojobori method. (C) Frequencies of normalized blast scores in pair-wise comparisons between Sheeba and three other genomes.

The size and GC content of the Sheeba genome are very similar to those of 26695 and J99 (Table 1), and all three genomes share numerous orthologous coding sequences (CDSs) (Figure 2). The proteins encoded by the 612 orthologous CDSs that are present in all three genomes and do not have internal gaps differ at only few of their amino acids, as indicated by pair-wise estimates of 3–4% for D_N_, the frequency of non-synonymous nucleotide polymorphisms (Table S1). Similarly, for most CDSs in *H. acinonychis,* normalized Blast scores against either of the two H. pylori genomes were very high (Figure 2C), whereas much lower scores were found in pair-wise comparisons against *Helicobacter hepaticus,* a distantly related *Helicobacter* species from rodents [18]. H. acinonychis is thus very closely related to *H. pylori,* almost as closely related as are the two H. pylori genomes to each other.

**Table 1:**
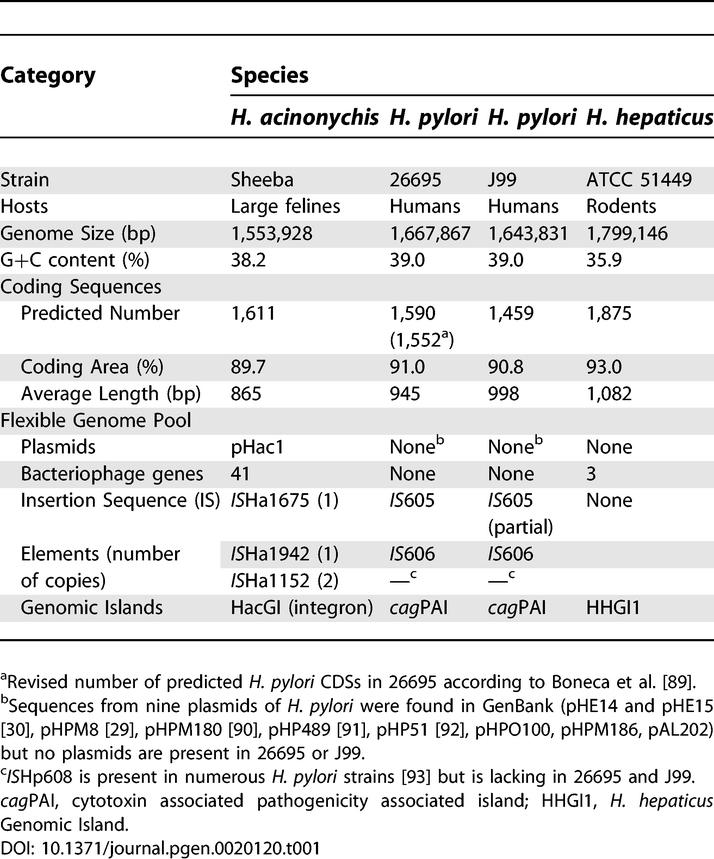
General Features of *Helicobacter* Genomes

D_S_, the synonymous genetic distance, can be used to estimate the elapsed time since divergence from a last common ancestor (LCA) of the three genomes. D_S_ was calculated among the 612 orthologs described above by two different estimators, that of Li et al. [19] and the modified Nei-Gojobori method [20], which is based on the ratio of transitions to transversions. Each method yielded different estimates but both methods indicated that D_S_ between Sheeba and either 26695 or J99 is approximately twice as high (2.0 ± 0.05) as D_S_ between 26695 and J99 (Figure 2B). This indicates that Sheeba diverged from the LCA two-fold as long ago as did 26695 and J99 from their last common ancestor. Thus, an estimate of the time of divergence between 26695 and J99 would automatically also provide an estimate of the divergence time for Sheeba from the LCA. Strain 26695 belongs to the hpEurope population of *H. pylori,* which is found throughout Europe and countries colonized by Europeans, while J99 belongs to hpAfrica1, which is found in Africa and countries affected by the slave trade [8,15]. The accumulation of sequence differences between hpEurope and hpAfrica1 has probably occurred over the same time scale as divergence within modern humans from their LCA, or approximately 100,000 y (100 ky) [21,22] (range based on molecular data: 50–200 ky [23–26]). This interpretation implies that 200 ky (range 100–400 ky) has elapsed since Sheeba diverged from the LCA (Figure 2B; see Materials and Methods).

### Genomic Differences between H. acinonychis and H. pylori

Having demonstrated that the core genomes of *H. pylori* and H. acinonychis are very similar, we now focus on genomic differences that might be relevant to a host jump. Gene products that become unimportant or even deleterious after a host jump might be lost or inactivated. Alternatively, a host jump may be triggered or facilitated by the acquisition of novel genes by horizontal gene transfer (HGT). In both cases, the absence or presence of such genes should be uniform within the newly evolved species, whereas genes that are variably present after a host jump probably reflect events that were not essential for that host jump because they arose subsequently. Therefore, we searched for genes that were specifically inactivated or novel in the genome of Sheeba versus 26695 and J99. To this end, when CDSs in one of the genomes were markedly shorter than their orthologs in the other genomes, the length of the longest orthologous CDS among the genomes was used to mark the boundaries of a “gene” and genes that contain one or more markedly shorter CDSs will be referred to as “fragmented.”

**Figure 3:**
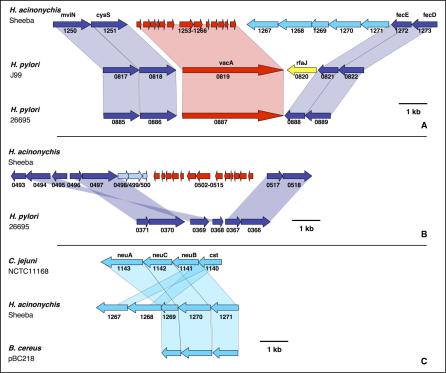
Gene Fragmentation, Duplication, and Import (A) Fragmentation of the *vacA* (vacuolating cytotoxin) gene (red) into 13 fragments and import of *neuACB, cst, cst* (blue-green) within Sheeba. *neuACB* encode acylneuraminate cytidyltransferases and *cst* encodes a sialyltransferase. (B) Translocation of a duplicate of the fragmented *vacA* gene to a different genomic location. The duplicated *vacA* gene (red) contains the same fragmentation pattern and differs by only one sequence polymorphism in 3,815 bp from that in part A, indicating that the duplication is recent and occurred after the fragmentation event. Next to the duplicated *vacA* gene are located three genes (light blue) that are unique to H. acinonychis. (C) Homologies of the *neuACB, cst, cst* gene cluster from part A with syntenic clusters in C. jejuni NCTC11168 and the B. cereus virulence plasmid pBC218.

The frequency of fragmented genes clearly indicates that the host jump proceeded from primates to large felines. Scattered around the Sheeba genome (Table S2) are 255 CDSs derived from 92 fragmented genes. Gene fragmentation accounts for more predicted CDSs, less coding area, and smaller average CDS size within the Sheeba genome than within 26695 or J99 (Table 1). Gene fragmentation was due to frameshift mutations and stop codons and only one of the 255 fragmented CDSs was associated with the transposition of insertion elements (ISs). Conserved hypothetical proteins, whose relevance to a host jump is uncertain, are encoded by 45 fragmented genes. The other frequent classes (Figure S2) encode 12 outer membrane proteins (OMPs), 11 restriction or modification enzymes, eight transport systems, six transposases, three fucosyl transferases, and two VacA vacuolating cytotoxins, that arose by a duplication event after the *vacA* gene was shredded into 13 fragments (Figure 3).

The frequency of fragmented genes is particularly high in Sheeba compared to the H. pylori genomes, even among the 1,306 CDSs that are common to all three genomes (Table 2): almost three times as many Sheeba genes are fragmented as in 26695 and over ten times as many as in J99. Indeed, 45 genes are fragmented in Sheeba but intact in both 26695 and J99, whereas only one gene is fragmented in both 26695 and J99 but intact in Sheeba.

**Table 2:**
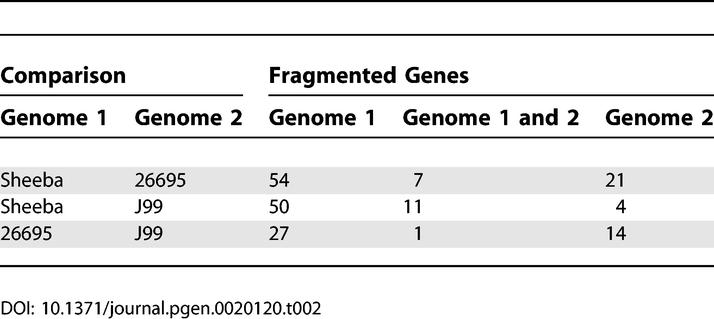
Numbers of Fragmented Genes among 1,306 Common Genes in Sheeba, 26695, and J99

**Figure 4:**
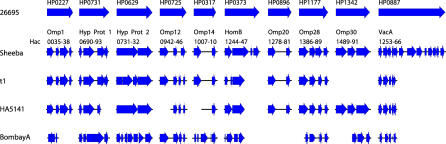
Fragmentation Patterns in Ten Genes among Three H. acinonychis Strains Ten genes that are intact in 26695 but are fragmented in the Sheeba genome (subgroup B) were re-sequenced from strains t1 and HA5141 of subgroup B and BombayA of subgroup A. Black lines indicate sequenced fragments and thick blue arrows indicate CDSs of ≥140 bp. Designations at the top indicate CDS designations in 26695 whereas designations above the Sheeba sequences indicate both the protein name and the CDS designations in Sheeba (Hac0035, Hac0036, etc.).

We tested whether fragmentation is uniform within H. acinonychis by re-sequencing ten fragmented genes from three additional strains of *H. acinonychis,* except for two PCR products that could not be amplified. All these sequences were fragmented (Figure 4), but they differed by single nucleotide polymorphisms and/or small deletions (Table S3). Two differing patterns of fragmentation were found that correspond to sub-groupings within H. acinonychis that were revealed by microarray analysis (see below). Thus, isolates of H. acinonychis from lions, tigers, and cheetahs all contain multiple fragmented genes that probably arose early after a host jump to large felines.

Similar to fragmented genes, the deletion of entire genes might possibly also have been associated with the host jump, especially because 144 genes are lacking in Sheeba that are present in both 26695 and J99 (Figure 2). However, according to DNA microarray analyses, hundreds of CDSs have been lost from at least some of 56 representative strains of H. pylori [15]. Thus, the absence of 144 genes within Sheeba versus 26695 and J99 need not reflect events that are relevant to or occurred soon after the host jump. Of the 1,150 genes that are uniformly present within *H. pylori,* only ten, encoding six hypothetical proteins, two OMPs *(omp6, omp19)* and other functions *(icfA, thiM)* were absent in all four strains of H. acinonychis that were tested [15]. Thus, if a loss of gene function accompanied the host jump, most such events are probably represented among the 92 fragmented genes in Sheeba.

### Unique Genes and HGT

Was the host jump to large felines facilitated by the acquisition of novel genes via HGT? Sheeba possesses 156 CDSs that are absent in 26695 and J99 (Figure 2), many of which were possibly imported by HGT (Figure S3). Prophages are unknown in H. pylori but 41 of the imported CDSs in Sheeba are within two prophages, called prophage I (11.6 kb) and prophage II (28.4 kb) (Figure S1, Table S4). Multiple CDSs in prophage II are orthologs of genes within a prophage of Campylobacter upsaliensis RM3195 that was isolated from a patient suffering from Guillain-Barré syndrome [27]*.* Prophages have been implicated in the acquisition of virulence [28] and genes in prophages I and II might potentially be relevant to host adaptation and specificity. However, most of the CDSs in these prophages encode hypothetical proteins whose relevance to infection by H. acinonychis cannot currently be evaluated.

The flexible gene pool in Sheeba includes one or two copies each of three distinct ISs, with homologies to *IS*605, *IS*606, and *IS*Hp608, respectively (Table 1). Sheeba also contains a 3,661 bp plasmid, pHac1 (Figure S4), with homologies to H. pylori plasmids pHPM8 [29] and pHel4 [30]. And three genes, *mccB, mccC,* and *repA,* that are present in H. pylori on plasmids pHPM8 and pHel4, form the “HacGI” integron that is flanked by two ISs within the Sheeba chromosome (*mccC* is intact but *mccB* and *repA* are fragmented).

**Figure 5:**
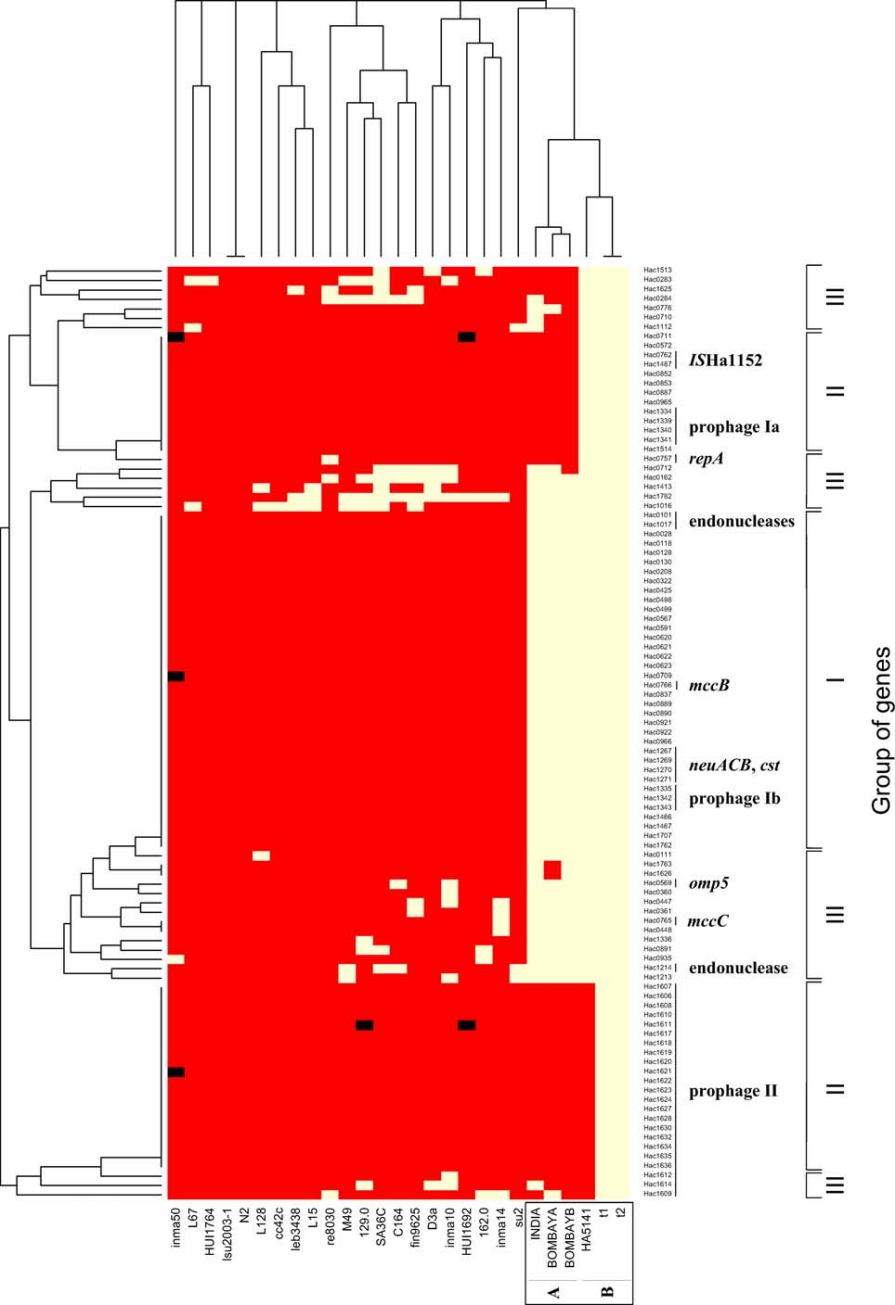
Hybridization of a DNA Microarray Chip Containing 99 PCR Products against Representative Strains of H. acinonychis and H. pylori Six strains of H. acinonychis from subgroups A and B and 21 strains that represent the genetic diversity of H. pylori (Table S5) were tested for hybridization (yellow) or lack of hybridization (red) with 99 PCR products (Table S6) from genes that are present in Sheeba and lacking in 26695 and J99. The results were clustered according to genes (left) and strains (top). Genes that hybridize exclusively with all H. acinonychis strains are summarized as group I (right), genes hybridizing only with some H. acinonychis are in group II and genes hybridizing with some H. pylori are in group III. Where gene functions were attributed, they are indicated at the right and other genes encode hypothetical proteins. black, missing data.

We tested whether these and other unique CDSs in Sheeba were universal within and specific to H. acinonychis by hybridization of six strains of H. acinonychis and 21 representative strains of H. pylori against DNA microarrays containing 99 PCR products (Figure 5). Only 37 CDSs were universally present within H. acinonychis and lacking in all H. pylori (group I). Others (group III) were also found within some strains of H. pylori and are not specific to H. acinonychis. Group III probably corresponds to genes that were present in a common ancestor and were subsequently lost in some H. pylori strains such as J99 and 26695 [15]. Still other CDSs (group II), including most of the prophage genes, were present within one subgroup of H. acinonychis but were lacking from the second. Group II genes are also unlikely to have been essential for the host jump to large felines and probably represent lysogenization and HGT that first occurred after genetic differentiation into subgroups.

The 37 CDSs in group I were probably also acquired by HGT. Most of them encode hypothetical proteins or are related to plasmid genes; the possible relevance of such genes for a host jump remains uncertain. However, group I does include a five-gene cluster, consisting of *neuACB* followed by two *cst* genes (Figure 3), all of which are in the same orientation. These are syntenic to homologous clusters of genes on the virulence plasmid pBC218 of Bacillus cereus [31] and the chromosome of *Campylobacter jejuni* NCTC11168 [32,33]. In both B. cereus and *C. jejuni,* these genes have been implicated in interactions with the host's immune response through sialic acid synthesis (*neuACB,* CMP-NANA synthetase, biosynthesis of N-acetyl-D-mannoseamine, NANA synthetase [34]) and transfer (*cst,* a bi-functional −2,3-/-2,8-sialyltransferase [33]) to carbohydrates on the bacterial cell envelope. Group I also included one Omp, *mccB,* and three genes from prophage I, all of which might be relevant to the host jump.

### Synteny and Genomic Rearrangements

**Figure 6:**
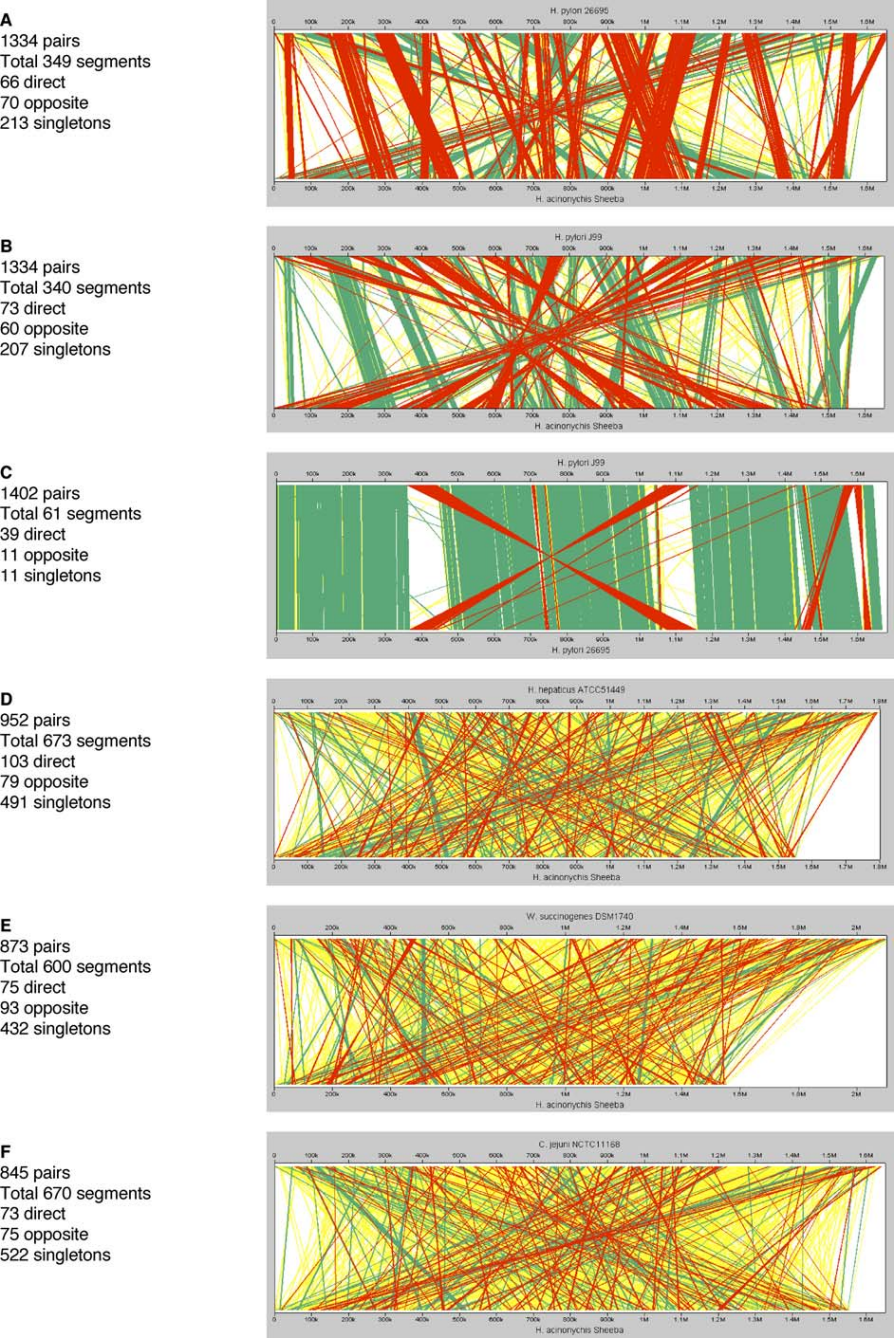
Genomic Rearrangements in Pair-Wise Genomic Comparisons between Sheeba and other *Helicobactera*ceae [16–18,85] or Campylobacteraceae [86] (A–B, D–F) or between 26695 and J99 (C) Stretches consisting of two or more syntenic orthologs are indicated in green for orthologs in the same orientation relative to the origin and in red for orthologs in inverted orientation. Single orthologous CDSs are indicated in yellow. Ortholog matches were plotted using CGViz (http://www-ab.informatik.uni-tuebingen.de/software/cgviz).

Large parts of the Sheeba genome are not only homologous to genes in 26695 and/or J99 (Figures 2 and S5) but are also arranged in syntenic order (Figures 6 and S6). Minor loss of synteny is due to the two prophages, ISs and plasmid-borne sequences that are specific to Sheeba (Table 1). Synteny is also disrupted by the absence of the 40 kb *cag* pathogenicity island (Figure S7), which apparently was acquired by H. pylori after the host jump to large felines that led to H. acinonychis [15].

The genomes of 26695 and J99 are largely colinear, with few exceptions [17], but only little colinearity is apparent in comparisons of H. pylori with the distantly related species *H. hepaticus, Wolinella succinogenes,* and C. jejuni [35]. Similarly, little obvious colinearity was detected when the Sheeba genome was compared to these distantly related species (Figures 6 and S6). In contrast, large genomic stretches were colinear between Sheeba and 26695 or J99, but these have been interrupted by at least 91 distinct genomic rearrangements.

Are these genomic rearrangements relevant to and did they possibly contribute to genomic adaptation during the host jump? In this case, one might expect to find a higher frequency of fragmented CDSs flanking the genomic rearrangements than the global frequency of fragmented CDSs within Sheeba. Alternatively, some or all of the rearrangements might have occurred within the lineage leading to H. pylori after the host jump. Most of the genomic rearrangements were flanked by intact housekeeping or hypothetical genes (Table 3) and we could not discern any obvious features of these flanking genes that could have provided an evolutionary or mechanistic basis for the rearrangements. However, 20 of the 182 flanking genes were fragmented within *H. acinonychis,* mostly corresponding to OMPs or restriction/modification enzymes. These genomic rearrangements may then have provided a mechanism for those instances of fragmentation and might be related to the host jump. Other genomic events such as duplication and gene conversion may also have resulted in several novel OMPs of the Hop and Hor family (Figure S8), which seem to represent mosaics between OMPs that are present in 26695 and/or J99. Thus, genomic rearrangements may have been an important mechanism for the inactivation of OMPs and their structural diversity. However, the overall frequency of fragmented CDSs flanking genomic rearrangements (11.6%) is lower than the frequency of fragmented CDSs within the Sheeba genome (16%), indicating that genomic rearrangements are not a major cause of gene fragmentation. Thus, many of the rearrangements are probably irrelevant to the host jump and may have occurred within the H. pylori lineage after its separation from the lineage leading to *H. acinonychis.*

**Table 3:**
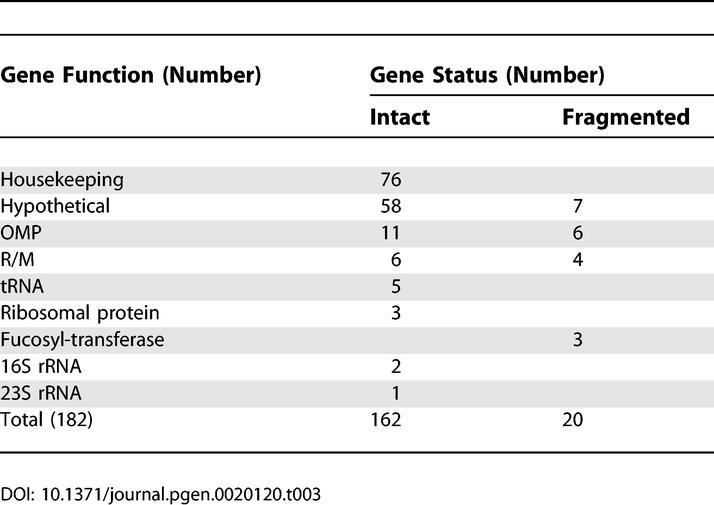
Genes in the Sheeba Genome Flanking 91 Genomic Rearrangements

## Discussion

### Evolution of H. acinonychis

Based on its ability to infect laboratory and captive animals, H. pylori infection can be thought of as an anthroponosis, whereby human infections are a source of disease for animals. We propose that just such an anthroponotic infection resulted in a host jump followed by speciation of H. acinonychis. According to this interpretation, H. acinonychis probably arose after a large feline became infected by eating an early human whose stomach was colonized by *H. pylori.* Such transmission events will only rarely result in host jumps, due to inefficient transmission of the pathogen between individuals of the novel host [36]. However, the particular transmission event that resulted in H. acinonychis was successful because the bacteria had imported novel genes and adapted to their feline host by gene inactivation. With time, the predecessor of H. acinonychis became so strongly adapted to infection of large felines that H. acinonychis now colonizes mice only poorly [5], and is not known to naturally infect any host species other than large felines. Random genetic drift after the host jump has also resulted in sub-differentiation into two genetic sub-groupings within this species.

A single host jump has been the source of all modern isolates of *H. acinonychis.* Otherwise, gene fragmentation patterns and the presence of imported genes would not have been as uniform as was observed*.* This conclusion contrasts with HIV viruses, where multiple host jumps from distinct species of chimpanzees have been inferred from phylogenetic analyses [37]. Similarly, multiple host jumps to humans are thought to have occurred with dengue viruses [38] and the possibility that additional host jumps from avians to humans may result in new influenza pandemics has been a recent cause of concern [36]. If H. pylori jumped from humans or other primates to large felines on multiple occasions, those bacteria are not included within *H. acinonychis,* which is monophyletic, but might instead be found among the non-cultivatable *Helicobacter* that have been described in the stomachs of large felines [12].

### When Did It Happen?

Several bacterial pathogens are thought to have evolved recently. These include *Yersinia pestis,* a clone of Yersinia pseudotuberculosis which has differentiated within the last 20 ky [39], *Mycobacterium tuberculosis,* a clone of smooth tubercle bacilli that diversified over approximately the same time period [40,41] and Salmonella enterica Typhi, a clone of S. enterica that is thought to be approximately 50 ky old [42]. All of these dates are based on a molecular clock rate (5 × 10^−9^ per year) for synonymous mutations that is calibrated by the 140 My since Escherichia coli and S. enterica shared a common ancestor [43], an estimate that is now almost 20 y old. Unfortunately, the synonymous molecular clock rate is not necessarily constant for different bacterial species and its ratio to the 16S rRNA clock rate can vary by an order of magnitude [44]. The currently most reliable bacterial synonymous clock rate is probably that for the obligate intracellular bacteria, Buchnera aphidicola (8 × 10^−9^) [44], which was calibrated by the dates of separation of their insect hosts based on fossil data. Not only are all other clock rate estimates almost certainly much less reliable, but it is also not clear whether clock rates based on times of separations of 50–150 My can even be extrapolated to much more recent events.

Based on synonymous differences over 612 conserved genes that are present in all three genomes, H. acinonychis diverged from H. pylori twice as long ago as did hpEurope from hpAfrica1. Our interpretation that hpEurope separated from hpAfrica1 100 ky (range: 50–200 ky) ago then extrapolates to an estimate of 200 ky (range: 100–400 ky) for the age of H. acinonychis. The time of separation between hpEurope and hpAfrica1 was equated with the time of separation between modern Africans and Europeans [21–26], which is justified by the close correlations between genetic diversity in H. pylori and ancient human migrations [8]. However, the time of separation between Africans and Europeans is not known with certainty, and may well be modified as additional evidence becomes available. Furthermore, rather than using the distance between one representative of each of the bacterial populations, it would have been preferable to base time estimates on genetic distances between the entire hpEurope and hpAfrica1 populations, after correcting for admixture and drift. It would also have been desirable to calibrate the clock rates after incorporating additional milestones in human history that can be associated with separation into distinct bacterial populations. Because neither of these improvements is currently feasible, we calculated an upper limit on the time since the most recent common ancestor of H. pylori plus H. acinonychis based on the 3,406-bp concatenated sequences in Figure 1. The Bayesian Skyline coalescent model [45] that is incorporated in Beast indicated a maximal age of 395 ky (95% confidence limits: 347–447 ky), which supports our initial estimate of the age of H. acinonychis. However, we note that coalescent analysis is inappropriate for recombining sequences, such as in *H. pylori,* and therefore feel that these upper limits should be treated with caution.

When taken at face value, our estimate of 100 ky for the separation of hpEurope from hpAfrica1 indicates that the synonymous molecular clock rate is 6.2 × 10^−7^ [19] to 9.2 × 10^−7^ [20], depending on the algorithm used to calculate synonymous distances, values that are 50-fold higher than the synonymous clock rate for E. coli – *S. enterica.* Higher mutation rates in H. pylori than within enteric bacteria are not necessarily surprising: the MutS mismatch repair system that reduces the mutation frequency in E. coli is not functional in H. pylori [46,47] and unlike *E. coli,* where mutators in mismatch repair are rare among natural isolates [48], many natural isolates of H. pylori have high mutation rates [47]. These considerations also support the inference by Ochman et al. [44] that different bacterial species may possess very different synonymous clock rates.

### Genomic Changes Related to the Host Jump

Comparative genomic analyses of several pathogenic bacteria have demonstrated that niche changes and host specialization can be accompanied by an explosive multiplication of insertion elements plus an accumulation of frameshift mutations and stop codons, which result in genome degradation [49–52]. However, in almost all cases, it is very difficult to distinguish genomic differences that accompanied the niche change from events that reflect subsequent microevolution within a species. For example, microevolution within Y. pestis has resulted in genomic rearrangements [53] that are extensive even in comparison with the genomic differences between Y. pestis and Y. pseudotuberculosis [51]. Similarly, major differences in genomic content were found by comparisons of multiple strains within E. coli [54] and Streptococcus agalactiae [55]. Given these observations, we attempted to identify genomic changes related to the host jump by focusing on differences between H. pylori and H. acinonychis that were unique to and ubiquitous throughout the latter. We therefore ignored genes that are specifically lacking in the Sheeba genome relative to 26695 and J99, because almost all these genes are also lacking in some strains of H. pylori [15]. Similarly, we ignored prophages and other genes that are present in some isolates of H. acinonychis but lacking in others. Such genetic changes may be interesting for an understanding of microevolution within *H. acinonychis,* but they are almost certainly unrelated to a host jump that only happened once. Finally, many of the genomic rearrangements that distinguish H. pylori from H. acinonychis may have occurred during the evolution of H. pylori 100–200 ky ago, before the separation of hpEurope from hpAfrica1, rather than within *H. acinonychis.* Instead, our focus on the host jump identified a limited number of genes that fulfilled our criteria: similar to Chlamydophila abortus [52], genes in Sheeba were fragmented due to frameshifts and/or stop codons (but not insertion elements as in Bordetella pertussis [50] or Y. pestis strain CO92 [56]) or had been imported from unrelated bacteria by HGT. A small fraction of the fragmented genes were also associated with genomic rearrangements. The frequency of fragmented genes in Sheeba is unusually high: 16% of the CDSs in the Sheeba genome were derived from fragmented genes (Table 1), whereas lower frequencies were observed in B. pertussis (9.4%), Y. pestis (3.7%), and *Cp. abortus* (3.0%), which are also thought to have undergone gene fragmentation due to a change in niche specificity.

Most of the genes putatively associated with the host jump lacked known homologs (“hypothetical proteins”). It is quite likely that some of these are relevant to niche adaptation, as documented on several occasions in other microbes [57]. However, it was particularly striking that genes that are potentially linked to changes in the bacterial cell surface were so prominent among the fragmented and imported genes, suggesting that changes of the bacterial cell surface were essential for the success of the host jump to large felines. H. acinonychis contains an imported *neuACB,cst,cst* cluster of genes that can potentially sialylate the bacterial cell surface polysaccharides and evade host immune defenses. Sialylation of lipopolysaccharide renders Neisseria gonorrhoeae resistant to complement-mediated phagocytosis [58] and has been invoked as a virulence and host-specificity determinant for pathogenic *Neisseria* and Haemophilus influenzae [59]. Similarly, 12 OMPs are fragmented and two others are specifically lacking within the Sheeba genome. Host immunity drives sequence variation and lack of expression of OMPs in Neisseria meningitidis [60,61] and variable expression of OMP lectins is important for the host-specific adaptation of H. pylori [62,63]. Thus, fragmentation and novel combinations of OMPs in H. acinonychis may have eliminated cell-surface targets for immune defenses and provided novel adhesins that were important for the adaptation to its new host.

### Subsequent Genomic Changes

Once adaptation to its initial host had succeeded, H. acinonychis spread globally within and between distinct feline species. Today, H. acinonychis can be isolated from cheetahs, lions and tigers, who differentiated from their LCA 11 million y ago [64], long before the host jump. During its global spread, two subgroups evolved within H. acinonycis with distinct gene content (Figure 5), sequence polymorphisms [5,15] and fragmentation patterns [5] (Figure 4). Possibly, the two subgroups are specific for different species of large felines in nature, but this question cannot currently be resolved because all currently available isolates of H. acinonychis are from captive animals. Subgroup B, including Sheeba, carries two prophages that are largely absent from subgroup A. We note that the acquisition of prophages and HGT may have been facilitated by changes in the complement of restriction/modification enzyme systems that can defend against the acquisition of foreign DNA. Due to extensive gene fragmentation, Sheeba contains only six potentially functional restriction/modification enzyme systems, consisting of three orthologs of the eleven that are found in H. pylori 26695 [16] plus three novel restriction endonucleases (CDSs Hac0101, Hac1017, Hac1214).

### Concluding Remarks

Zoonoses resulting in potential human epidemics have recently received considerable attention [36,37,65]. Possibly due to our anthropomorphic bias, the contrary phenomenon of an anthroponosis resulting in a host jump to an animal has received much less attention. The natural infection of armadillos by Mycobacterium leprae probably represents one such example of an anthroponotic host jump [66] and the infection of large felines by H. acinonychis provides a second. In both cases, isolates from humans strongly resemble those from the novel host, indicating that only very few changes may be needed for such a host jump to succeed. DNA can readily be transferred by transformation between H. pylori and H. acinonychis [67], and H. acinonychis can infect laboratory animals [5]. The genomic sequence described here now provides the prerequisites that can allow the dissection of the molecular basis of host jumps in bacterial pathogens.

## Materials and Methods

### Bacterial strains.

Bacterial strains are listed in Table S5. Strain Sheeba was isolated from a biopsy of a Russian circus lion with gastroenteritis. It was chosen for sequencing because its genome was the largest among the seven isolates of H. acinonychis in Table S5 according to pulsed-field gel electrophoresis.

### Genome sequencing and annotation.

Genomic DNA from H. acinonychis Sheeba was isolated using the Qiagen Genomic DNA Kit (Qiagen, Hilden, Germany). Shotgun DNA libraries with insert sizes of 1–2 kb, 3–5 kb (TOPO Shotgun Subcloning Kit, Invitrogen, United States) and 40 kb (EpiFOS, Epicentre, Madison, United States) were end-sequenced to 7-fold coverage and remaining gaps were closed by direct sequencing, using genomic DNA as a template. The final sequencing error rate was estimated to be <2 × 10^−6^ using the Phred/Phrap/Consed software package [68–71].

Curation and annotation of the genome was performed using the annotation package GenDB [72]. Protein coding genes were predicted with the programs Glimmer [73] and Critica [74], which are integrated into the GenDB package, and annotated on the basis of similarity searches against public databases using Blastx and manual curation. The remaining ORFs were post-processed with RBSFinder [75], which identifies ribosomal binding sites. Functional classification to COG categories [76] was performed with InterPro and metabolic pathways were constructed with reference to KEGG [77]. The origin of DNA replication was localized by identification of known motifs of the primosome as well as the GC skew of the genome. tRNA and rRNA genes were identified by tRNA-Scan and Blastn, respectively.

All predicted CDSs of the three species were tested in reciprocal pair-wise Blastp comparisons using a cut-off e-value of ≤1 × e^−15^, followed by manual editing. Fragmented, paralogous or duplicated CDSs were each assigned a distinct CDS number (Hac0001, Hac0002, etc.). As a result, Sheeba contains more CDSs than the total numbers of shared and species specific genes in Figure 2. A list of the unique genes within Sheeba can be found in Table S4.

### Sequence homologies between genomes and age calculations.

Blastp analyses were performed for each translated CDS within the Sheeba genome against itself and against a query genome. The normalized blast score is the ratio of the score for the query genome divided by the self score.

In order to calculate pair-wise genetic distances for age calculations, we compiled a list of 1,058 unfragmented orthologs with reciprocal best Fasta hits of >80% homology that were present within Sheeba, 26695 and J99. 612 of these orthologs contained no internal gaps and were used to calculate synonymous differences. After excluding the start and stop codons, the number of potential synonymous sites and non-synonymous sites were calculated separately according to the algorithms of Li et al. [19] and the modified Nei-Gojobori algorithm, which accounts for R, the ratio of transitions to transversions (see pages 57–58 of Nei and Kumar [20]) (Table S1). The number of synonymous changes in pair-wise comparisons was calculated as described on pages 52–54 of Nei and Kumar. Synonymous distances were calculated after applying the Jukes and Cantor correction for forward and reverse mutations [78] by the formula


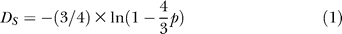


where *p* is the observed proportion of synonymous changes and D_S_ is the corrected synonymous distance.

In order to calculate the time since a last common ancestor, we calibrated the synonymous molecular clock rate on the basis that genomes 26695 and J99 belong to the hpEurope and hpAfrica1 populations, respectively, whose human hosts diverged ~100 ky ago [21,22], for a total separation time of ~200 ky. This calibration results in a synonymous clock rate (rate) of 9.23 × 10^−7^ [19] to 6.13 × 10^−7^ (modified Nei-Gojobori) per year. Assuming a constant clock rate, D_S_ between Sheeba and 26695 or J99 can be used to calculate the time since their last common ancestor *(age)* as


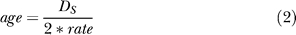


*age* is 205,471 ± 271 y according to Li et al. [19] and 196,169 ± 244 y according to the modified Nei-Gojobori method, for a mean value of 200,820 ± 258.

### DNA microarray analyses.

Microarrays were constructed and analyzed as described [15], with the following differences. The microarray chip contained six copies of 99 unique CDSs plus seven housekeeping genes *(atpA, efp, mutY, ppa, trpC, ureI,* and *yphC),* amplified from two strains each as a positive control, and printing buffer as a negative control. PCR products corresponding to at least 80% of the target CDS were amplified using specific amplification primers that were designed using Primer3 [79] (Table S6). The microarrays were hybridized against fluorescently labeled (Cy3/Cy5) genomic DNA from six strains of H. acinonychis and 21 strains that represent the global diversity of H. pylori (Table S5) with dye-swapping. Local background values were subtracted from each spot, the intensities for each fluorescent signal were normalized to the geometric mean intensities of the positive controls, and the geometric means of the intensities of the six copies per chip were calculated. Individual fluorescence values that deviated from the geometric mean by more than three-fold were excluded from the analysis and only geometric means based on at least two values were retained. These were then normalized to the geometric means of data from the housekeeping genes to yield a normalized ratio. The normalized means from the two dye-swap experiments were averaged, except in those few cases where only one normalized mean was available. Experiments in which both normalized means were lacking were treated as missing data. A histogram of the normalized ratios was used to determine a cut-off value of 0.4, above which hybridization with CDSs was scored as being present. This cut-off value reflects a local minimum within the histograms as well as the results of comparisons of the microarray data to manual inspection of the (unpublished data) genome sequence of H. pylori strain 162.0. Clustering of genes and strains was performed using the “heatmap.2” function of the package “gplots", that is implemented in R [80].

### Re-sequencing of ten fragmented genes.

The universality of fragmentation of genes within Sheeba was tested by re-sequencing ten genes from three additional isolates of H. acinonychis from PCR products that were amplified with the oligonucleotide primers in Table S7. The fragmentation patterns in Figure 4 are described in detail in Table S3. Strain BombayA (subgroup A) showed fragmentation patterns that differed extensively from those of Sheeba (subgroup B) while the fragmentation patterns of strains t1 and HA5141 (subgroup B) were similar to those of Sheeba.

### Phylogenetic analyses.

The sequence data in Figure 1, consisting of concatenated fragments of the *atpA, efp, mutY, ppa, trpC, ureI,* and *yphC* housekeeping genes (http://pubmlst.org/helicobacter) from 62 strains, have been described by Gressmann et al. [15]. Modeltest 1.05 [81] was used to determine that the optimal evolutionary model was GTR+G+I (parameters: gamma [shape] = 0.52; I [PInvar] = 0.62), which was then used to construct a neighbor-joining tree in PAUP* [82].

The maximal age of the most recent common ancestor (tmrca) of H. pylori plus H. acinonychis was calculated on the same data from the 62 strains using Beast V1.3 (http://evolve.zoo.ox.ac.uk/beast) after 10 million Markov chain iterations with sampling at intervals of 1,000. Similar results were obtained with concatenated sequences of all seven housekeeping gene fragments and of only the *atpA, efp, mutY,* and *ppa* genes. Similar results were also obtained when the tmrca was calculated including C. jejuni as an outgroup for concatenated sequences of the latter four gene fragments. It was not possible to test concatenates of more than four housekeeping genes with an outgroup because orthologs of *trpC, ureI,* and *yphC* are lacking in *H. hepaticus, W. succinogenes,* and C. jejuni. Finally, comparisons of the step-wise (“constant”) and linear population growth models also yielded in significantly different log likelihoods for the constant model and comparable results were obtained after specifying either three or ten populations. The results presented here are based on three populations, the linear growth model, seven housekeeping genes, no outgroup and a molecular clock rate of 2.32 × 10^−7^, which was calculated as above on the basis of all polymorphisms between 26695 and J99 in Table S1.

## Supporting Information

Figure S1 Circular Representation of the H. acinonychis Sheeba Genome Circular wheels from outside in: 1,2) CDSs on the plus (wheel 1) and minus (2) strands, colored according to COG classifications (see Figure S2); 3) fragmented genes indicated in red; 4) genes that are present in Sheeba but absent in both 26695 and J99. Dark blue, genes with database orthologs (Sept. 2005); light blue, genes without significant database homology; 5) genes associated with genomic mobility: orange = prophages, yellow = plasmid-associated, green = IS elements, light orange = restriction/modification; 6) GC plot, showing that genes in wheels 3–5 often deviate in GC content; 7) cumulative GC skew, (range: white [minimum] to black [maximum], calculated with Genskew [http://mips.gsf.de/services/analysis/genskew]), which was used to show that the origin of replication is near *dnaA*; 8) 36 tRNA genes (brown), two 16S rRNA, and two 23S-5S rRNA ribosomal regions (orange) are indicated by arrowheads.
(2.3 MB EPS)

Figure S2 Functional Classification of the Complete Genome Inventory of CDSs (A) and Fragmented Genes (B) of H. acinonychis Sheeba The pie charts show the frequency of CDSs by COG category [76], except for 39% of the CDSs that are indicated as “No hits” because hit levels were <1 × e^−15^ or they matched COG entries that were not yet assigned to a COG category. (A) All CDSs within the Sheeba genome were analyzed, including CDSs that were assigned to a fragmented gene. (B) Fragmented genes after reconstruction by comparisons with 26695 and/or J99. Eight of 31 genes within the “No hits” category represent OMPs, which are not assigned to a distinct COG category.
(41 KB PDF)

Figure S3 Taxonomic Sources of Closest Orthologs (A), Neighboring Mobile Elements (B) and Predicted Functions (C) of 93 Unique CDSs in Sheeba The following observations apply to 93 CDSs with database orthologs, and 63 other CDSs encoding hypothetical proteins without database orthologs were excluded. For most CDSs, the best Blast hits were to orthologs within the ɛ-Proteobacteria (A). Most unique CDSs are located next to prophages, plasmid-borne genes or IS elements (B), suggesting that they reflect HGT. However, the “core chromosome” genes neighboring 18 CDSs are homologous to genes in C. upsaliensis RM3195, C. jejuni NCTC1168, and H. pylori 99515 and might have been inherited by vertical descent.
(44 KB PDF)

Figure S4 The pHac1 Plasmid Six open reading frames on the plus (outer wheel, facing out) and minus (facing in) strands are color-coded according to COG classification as described in Figure S2. The gene *repA* (pHac06) is homologous to plasmid genes that are responsible for replication initiation. The N-terminal portion of *repA* is also homologous to Hac0757 on the Sheeba chromosome, which is located within the HacGI integron. Genes pHac01 and pHac02 are orthologs of jhp0828 and jhp0825, respectively, that flank an *IS*606 transposase (jhp0826–7) within the J99 chromosome. No database orthologs were found for three hypothetical genes (pHac03–05). The inner wheel shows cumulative GC skew, with color coding as in Figure S1. The average GC content of plasmid pHac1 is 34.6% versus 38.2% for the core chromosome. Plasmid pHac1 possesses homologies with the H. pylori plasmids pHPM8 (7.8 kb) and pHel4 (11.0 kb), including a perfect 22 bp repeat region upstream of *repA* gene, which is characteristic of theta plasmids [30,83].
(816 KB EPS)

Figure S5 Relatedness of Orthologous Genes between Sheeba and Other Genomes (A and B) and (D–G) show normalized blast scores of individual CDSs between Sheeba and other genomes, sorted by the CDS order within Sheeba, within which features of particular interest are highlighted by colors (see color code at the bottom). Cumulative normalized blast scores, sorted in descending order, are shown in (H). The normalized blast scores relative to H. pylori genomes in (A and B) were high for most CDSs, e.g. the highly conserved F0F1 ATP synthase or ribosomal proteins involved in translation. CDSs in (A and B) with low normalized blast scores are unique to Sheeba, and were attributed to HGT. These include the *neuACB*-*cst* cluster, two prophages, plasmid-associated genes and restriction and modification enzymes. Fragmentation within certain genes, e.g., *vacA,* was associated with a series of CDSs with intermediate normalized blast scores. Note that normalized blast scores against other Campylobacterales (D–F) were lower and against the unrelated Escherichia coli (G) were much lower than in comparisons with H. pylori (A and B). (C) is similar to (A), except that it shows a comparison between two H. pylori genomes and that most of the normalized Blast scores are high. Two arrows in (C) indicate hypothetical proteins flanked by the insertion sequence elements *IS*605, which correspond to regions 1 (left) and 3 of the five distinct G + C regions in 26695 [16].
(3.7 MB PPT)

Figure S6 Gene Order and Colinearity in *Helicobacteraceae* and *Campylobacteraceae* Genomes CDSs in genomic order were tested for colinearity between pairs of genomes, as indicated. Each point represents a matching pair of orthologs with an e-value of <1 × e^−15^ (A–F) or the maximal unique match ("MUM") (G–L) according to MUMmer [84] using 20 bp as the minimum length of a MUM. A summary of the genes flanking breakpoints in colinearity between Sheeba and 26695 or J99 is presented in Table 3. A quantitative co-linearity factor was calculated from the genomic positions (x and y coordinates) of each ortholog pair relative to O, the number of CDSs in the target genome, as follows. For each pair of neighbouring ORFs on the query genome (x_i_, x_i+1_), the position of the corresponding orthologs on the target genome (y_i_, y_i+1_) was used to calculate D = Min (|y_i+1_–y_i_|, O – |y_i+1_–y_i_|). The colinearity factor C = ΣD/O. These calculations yielded values for C of 18 for H. pylori J99 versus 26695, 41–45 for Sheeba versus 26695 or J99 and 204–238 for Sheeba versus H. hepaticus [18], W. succinogenes [85] or C. jejuni [86].
(863 KB PDF)

Figure S7 Genomic Content of the Region Flanking the *cag*PAI in H. pylori 26695 and J99 versus H. acinonychis Sheeba The *cag*PAI genes of H. pylori 26695 and J99 (red) are flanked by pairs of 31 bp repeats [87], as indicated by diamonds. The cagPAI region between the repeats is lacking in the Sheeba genome and only one of the repeats is present. The region flanking these repeats is syntenic in all three genomes, as indicated by color coding but has suffered some rearrangements. As part of these rearrangements, the syntenic cluster in yellow has been inverted and transposed downstream of the green cluster and additional sequences have been inserted into the grey region of Sheeba, resulting in a cluster of fragmented genes between Hac0887 and Hac0894, which shows a partial homology to HP0548 in 26695 and the region between jhp0495 and jhp0496 in J99.
(74 KB PPT)

Figure S8 Phylogenetic Tree of OMPs from the Hop and Hor Families in H. acinonychis Sheeba and H. pylori 26695 and J99 Omps of the Hop- and Hor- families [88] were assigned sequential numbers based on their genomic positions within 26695 (genes labelled HPxxxx) and J99 (genes labelled jhpxxxx) [16,17], except for HP1066 (HorD), which was designated OmpXX. We assigned independent, sequential numbers to OMPs within Sheeba based on their genomic locations within its genome; these numbers differ from those of orthologs in the H. pylori genomes due to genomic rearrangements. Orthologs between OMPs in the three genomes were determined by phylogenetic clustering of predicted proteins. Where pseudo-genes existed due to fragmentation, the clustering was based on reconstructed genes designated Hacxxxxrc for Sheeba and HPxxxxrc (Omp08, Omp03) for 26695. All Sheeba OMP genes have close orthologs within 26695 and/or J99, except for Omps 6 (Hac0713rc), 7 (Hac0751rc), and 8 (Hac0826) and all OMP genes within 26695 or J99 possess orthologs in Sheeba, except for Omps 1 (HP0009), 2 (HP0025), 6 (HP0229), 9 (HP0317), 13 (HP0638), 19 (HP0896), and 28 (HP1243). Of the Sheeba Omps that were reconstructed from gene fragments, seven (Omps 1, 12, 14, 20, 28, 30, 31) are of the Hop family, one (Omp18) is of the Hor family, and two (Omps 6, 7) are distantly related to both families. Dotted lines represent truncated numbers of nucleotide substitutions.
(46 KB PDF)

Table S1 Synonymous and Non-Synonymous Distances in Pair-Wise Comparisons of 612 Conserved Genes among Three *Helicobacter* Genomes (29 KB DOC)

Table S2 Fragmented Genes in Sheeba and Their Orthologs in 26695 and/or J99 (192 KB PDF)

Table S3 Sequence Differences of Ten Fragmented Genes between Sheeba and Three Strains of H. acinonychis
(67 KB PDF)

Table S4 Unique Genes in Sheeba (136 KB PDF)

Table S5 Sources of H. acinonychis and H. pylori
(56 KB DOC)

Table S6 Primer Pairs for Microarrays (43 KB PDF)

Table S7 PCR-Primer Pairs (86 KB DOC)

### Accession Numbers

The EMBL Nucleotide Sequence Database (http://www.ebi.ac.uk/embl) accession numbers used in this study include: Sheeba genome (AM260522), pHac1 plasmid (AM260523), and the sequences referred to in Figure 4 and Table S3, AM285650-77.

## Abbreviations

CDS: coding sequence
D_N_: pair-wise non-synonymous genetic distance
D_S_: pair-wise synonymous genetic distance
HGT: horizontal gene transfer
IS: insertion element
LCA: last common ancestor
OMP: outer membrane protein
