# Supplementary figures and images for: Who Ate Whom? Adaptive Helicobacter Genomic Changes That Accompanied a Host Jump from Early Humans to Large Felines
Source: PLoS Genet. 2006 Jul 28;2(7):e120. doi: 10.1371/journal.pgen.0020120 (PMC1523251; doi:10.1371/journal.pgen.0020120)

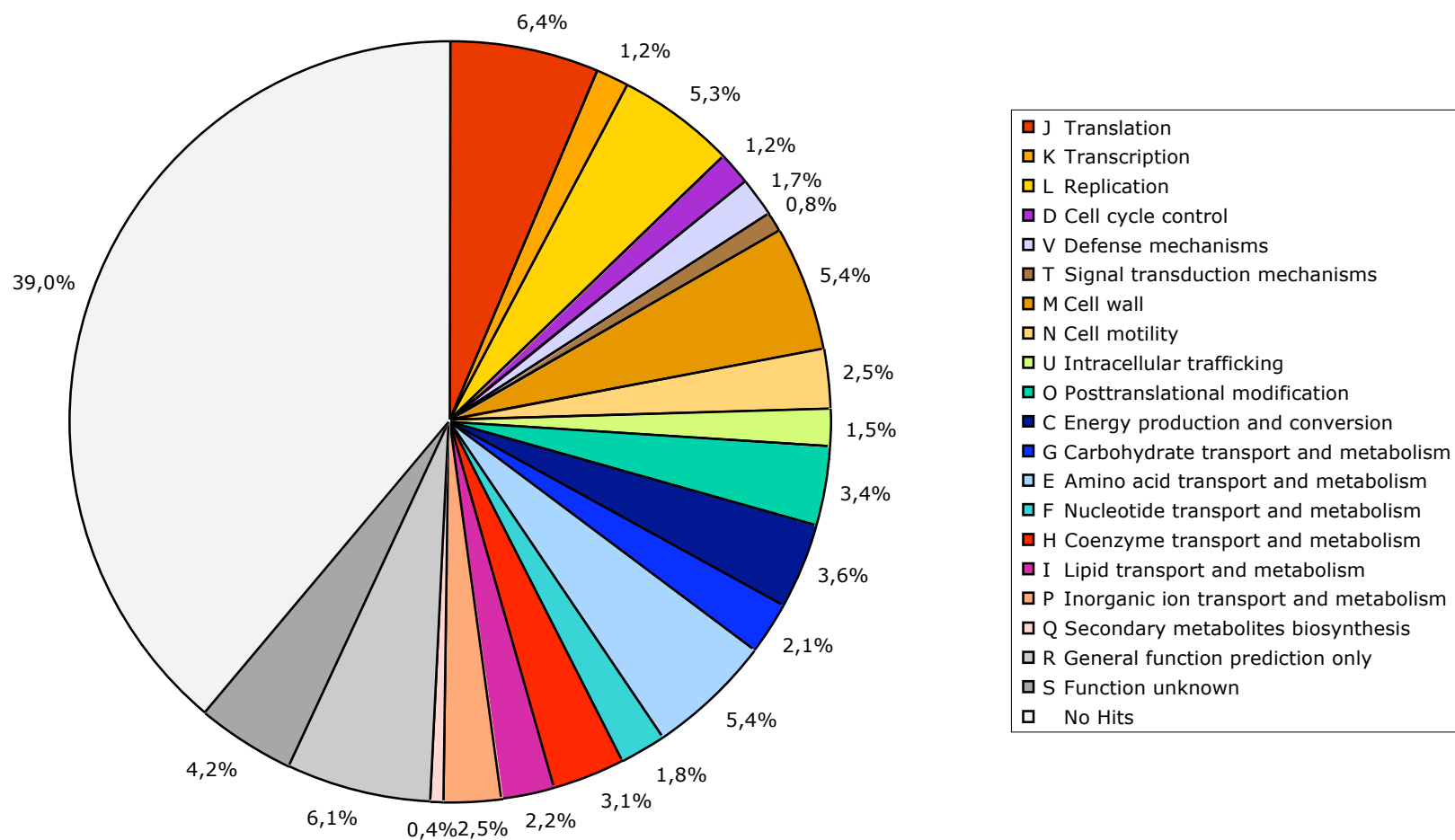

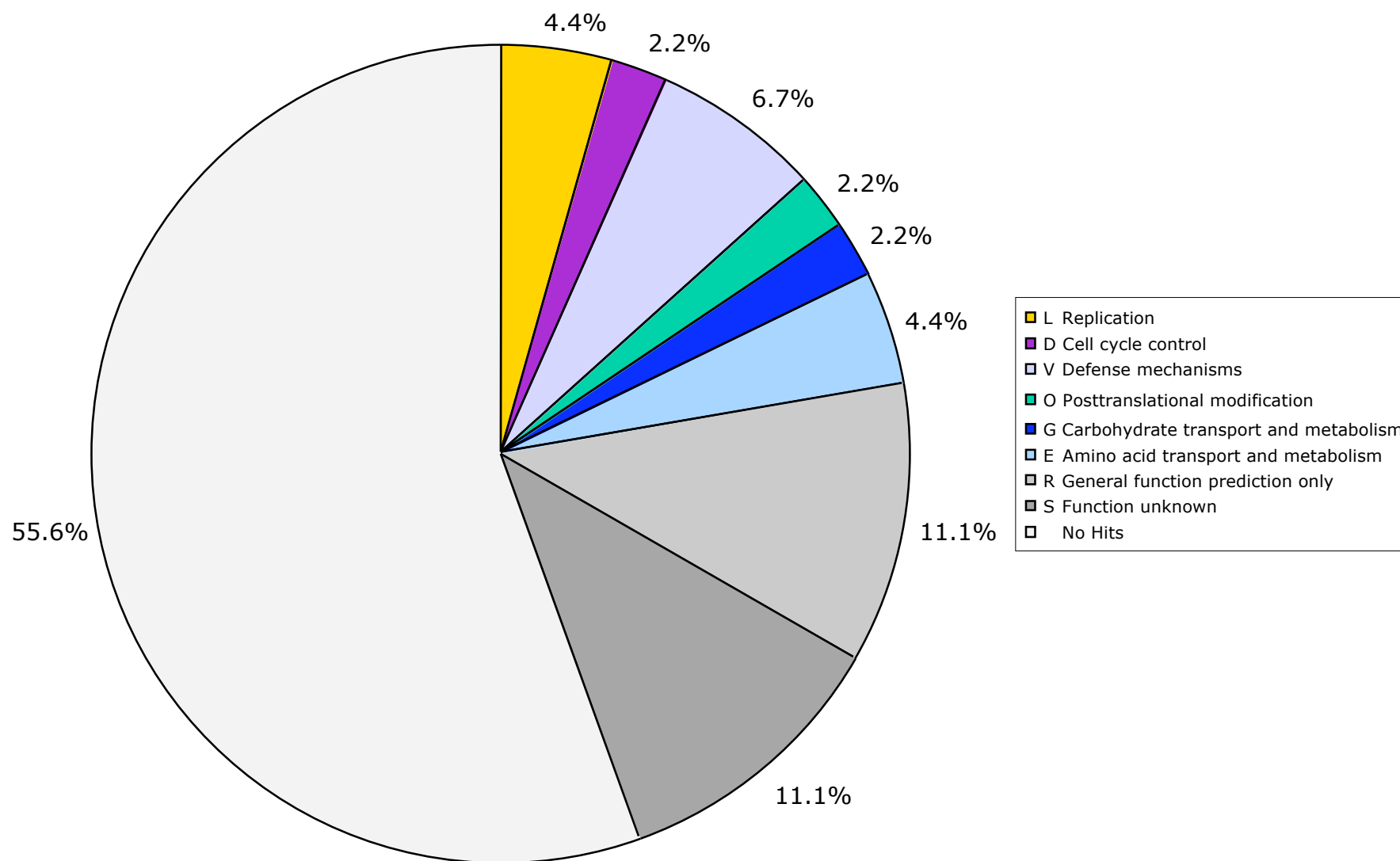

Supplement: Figure S2 — The pie charts show the frequency of CDSs by COG category [76], except for 39% of the CDSs that are indicated as “No hits” because hit levels were <1 × e−15 or they matched COG entries that were not yet assigned to a COG category. (A) All CDSs within the Sheeba genome were analyzed, including CDSs that were assigned to a fragmented gene. (B) Fragmented genes after reconstruction by comparisons with 26695 and/or J99. Eight of 31 genes within the “No hits” category represent OMPs, which are not assigned to a distinct COG category. (41 KB PDF) [file pgen.0020120.sg002.pdf]

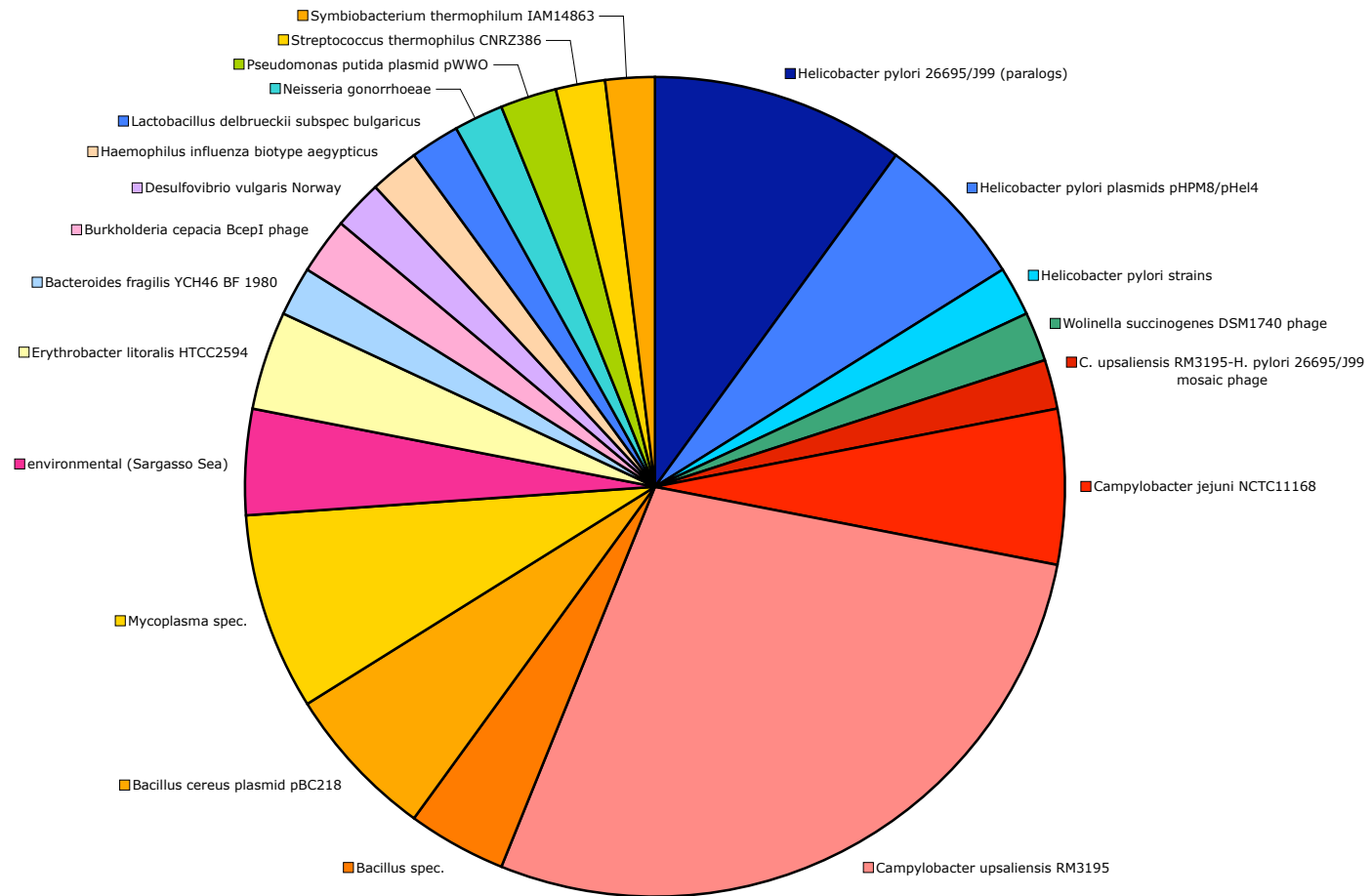

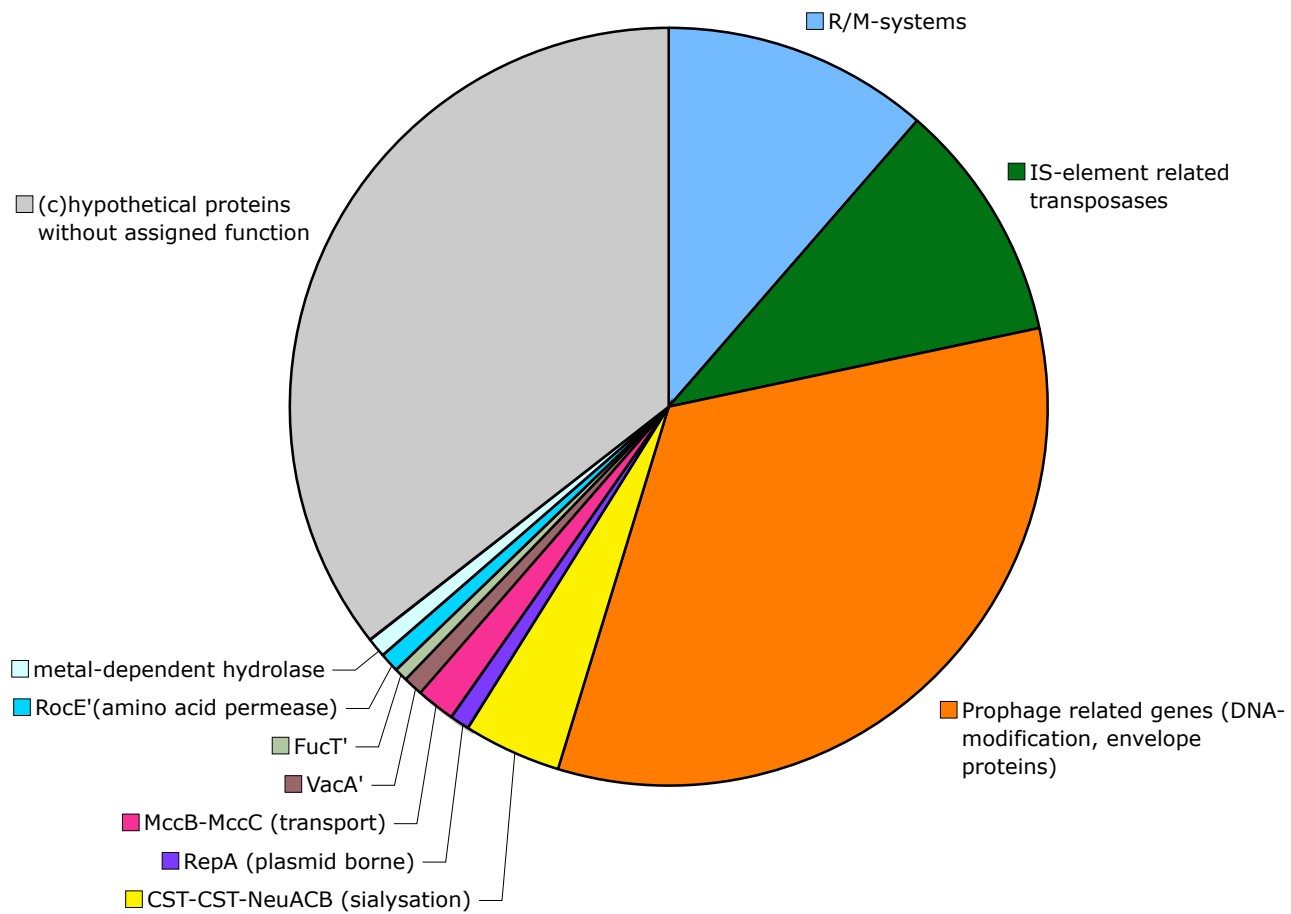

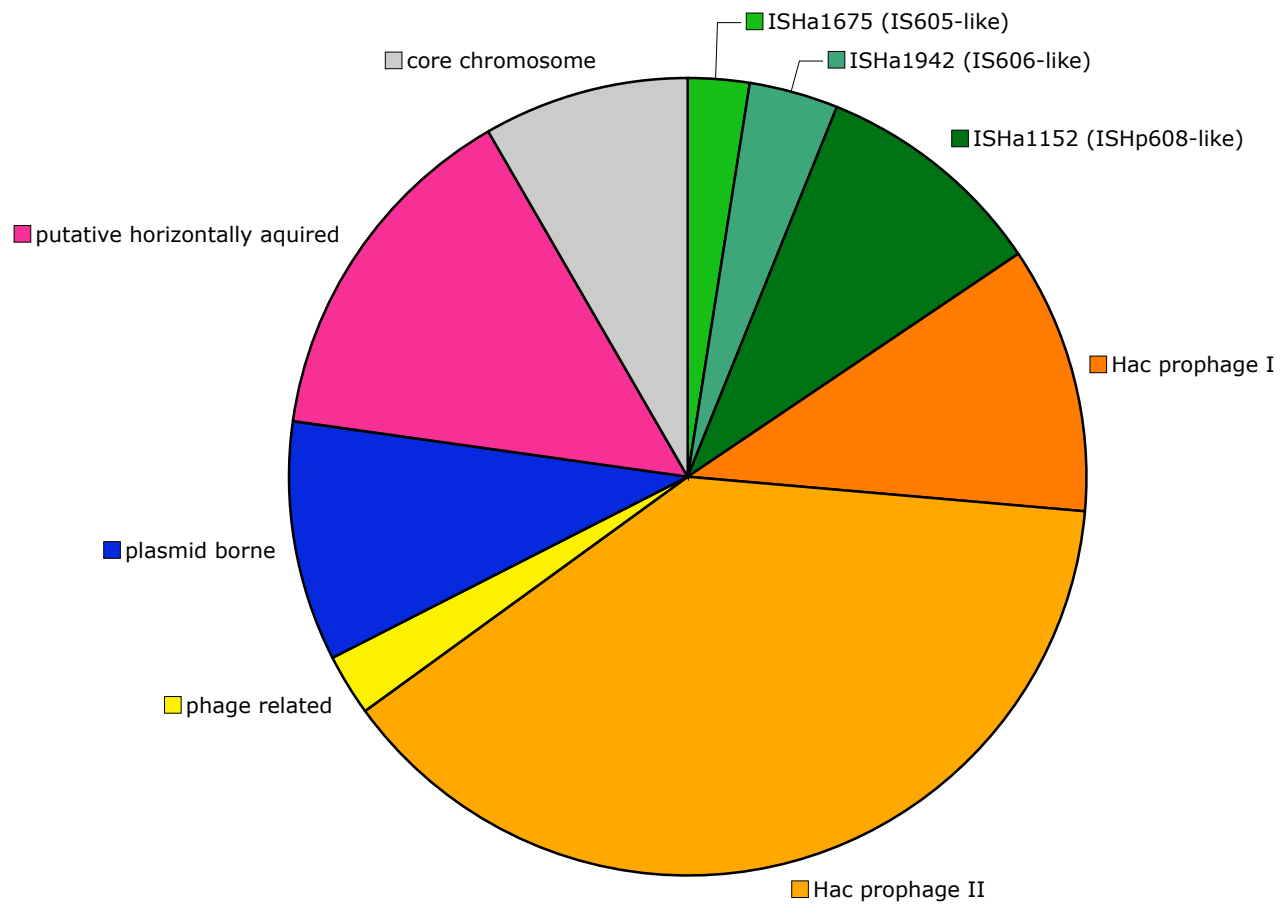

Supplement: Figure S3 — The following observations apply to 93 CDSs with database orthologs, and 63 other CDSs encoding hypothetical proteins without database orthologs were excluded. For most CDSs, the best Blast hits were to orthologs within the ɛ-Proteobacteria (A). Most unique CDSs are located next to prophages, plasmid-borne genes or IS elements (B), suggesting that they reflect HGT. However, the “core chromosome” genes neighboring 18 CDSs are homologous to genes in C. upsaliensis RM3195, C. jejuni NCTC1168, and H. pylori 99515 and might have been inherited by vertical descent. (44 KB PDF) [file pgen.0020120.sg003.pdf]

**A**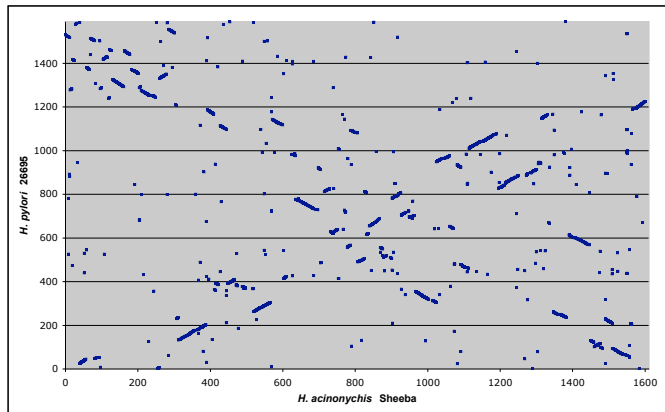**B**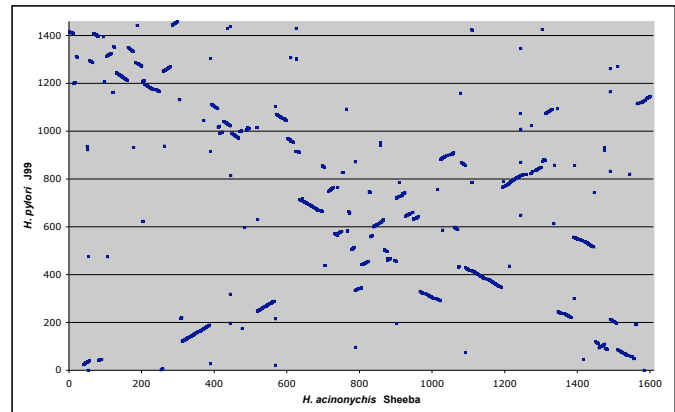**C**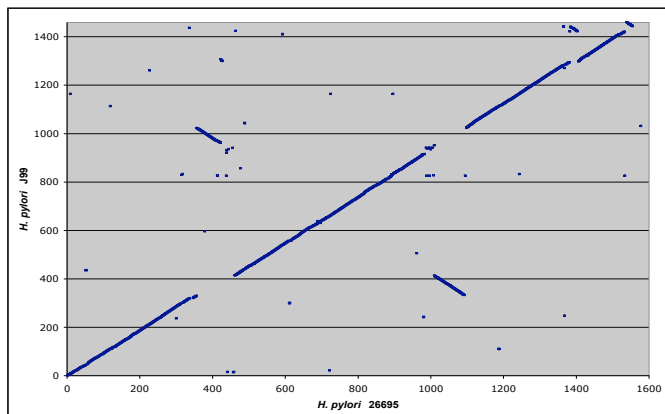**D**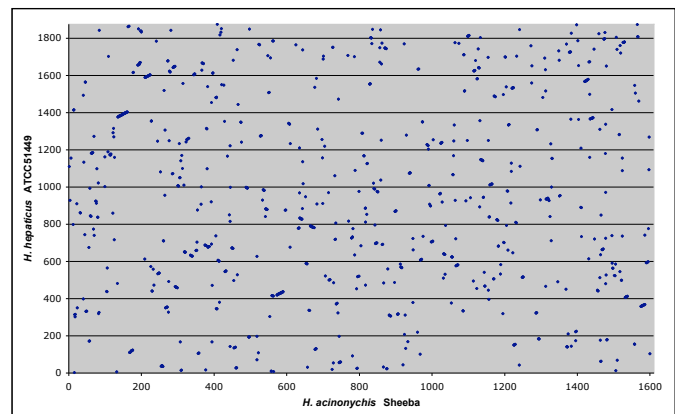**E**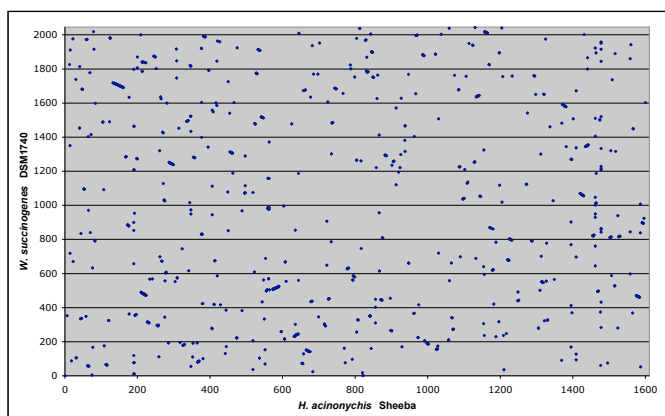**F**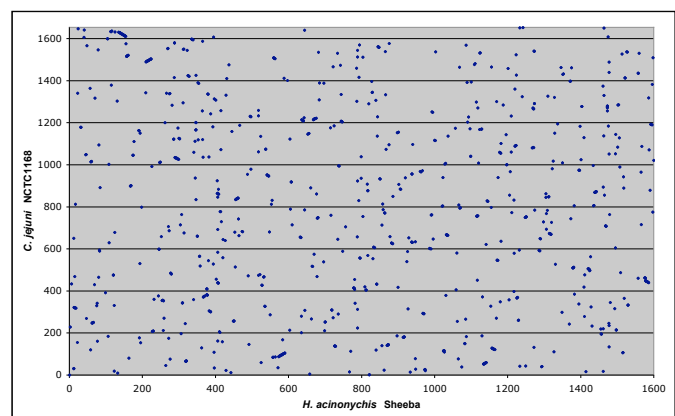

G

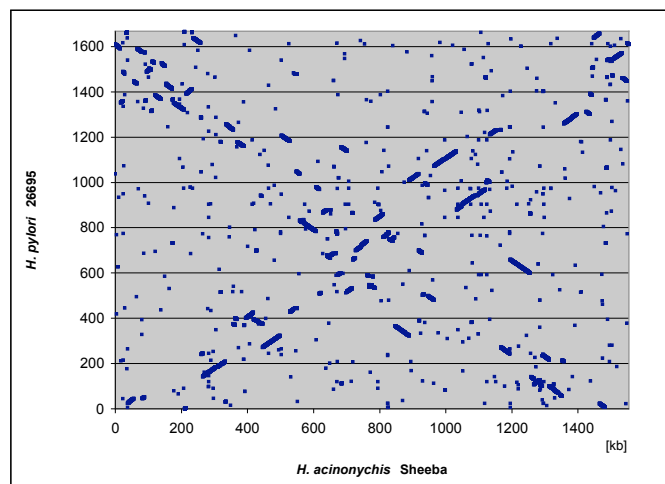

H

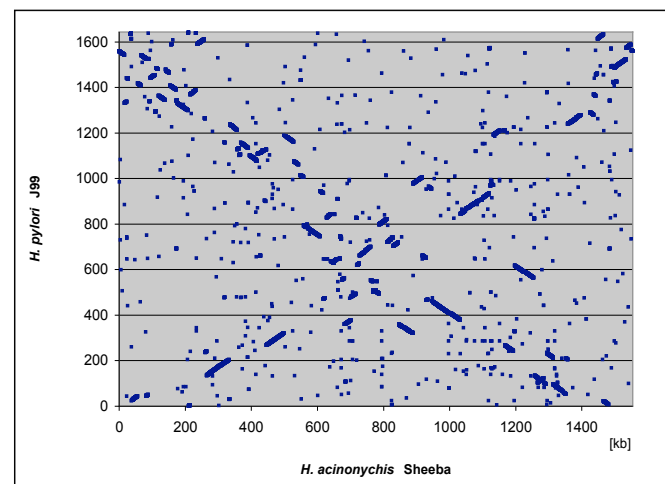

I

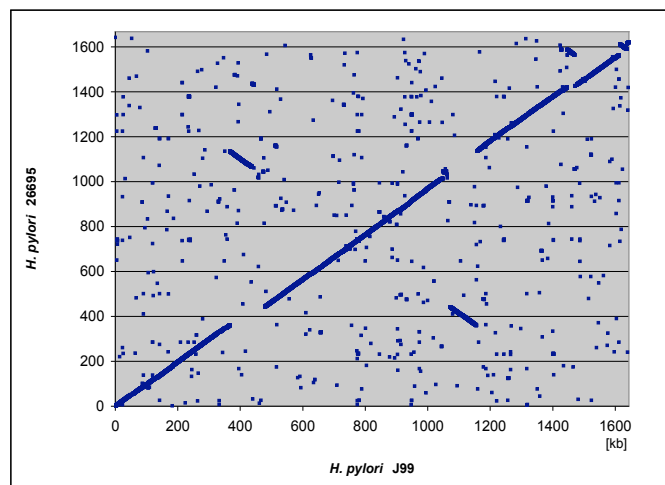

J

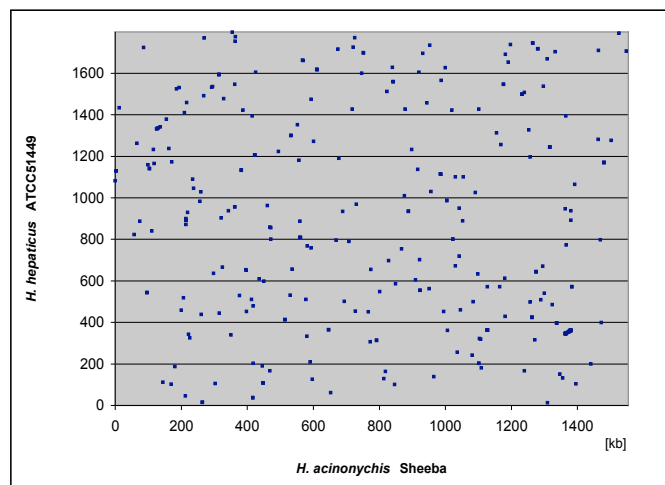

K

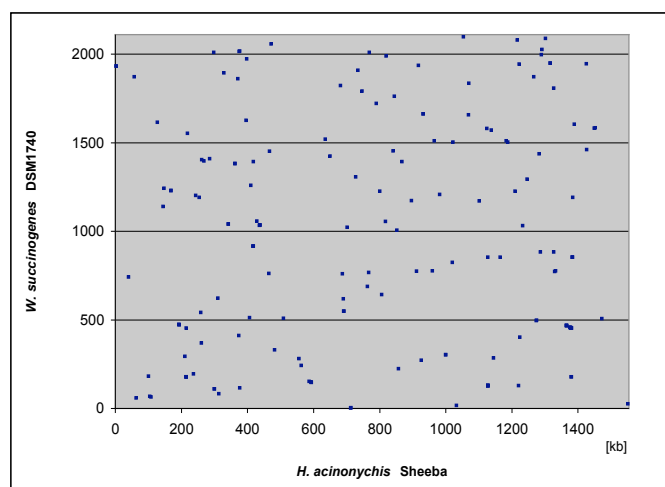

L

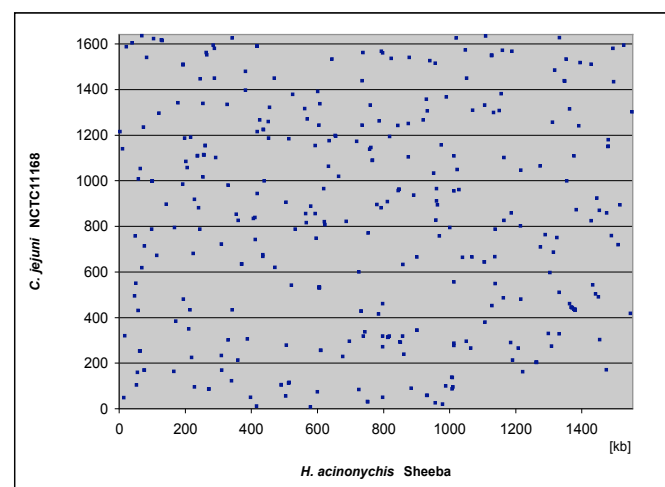

Supplement: Figure S6 — CDSs in genomic order were tested for colinearity between pairs of genomes, as indicated. Each point represents a matching pair of orthologs with an e-value of <1 × e−15 (A–F) or the maximal unique match ("MUM") (G–L) according to MUMmer [84] using 20 bp as the minimum length of a MUM. A summary of the genes flanking breakpoints in colinearity between Sheeba and 26695 or J99 is presented in Table 3. A quantitative co-linearity factor was calculated from the genomic positions (x and y coordinates) of each ortholog pair relative to O, the number of CDSs in the target genome, as follows. For each pair of neighbouring ORFs on the query genome (xi, xi+1), the position of the corresponding orthologs on the target genome (yi, yi+1) was used to calculate D = Min (|yi+1–yi|, O – |yi+1–yi|). The colinearity factor C = ΣD/O. These calculations yielded values for C of 18 for H. pylori J99 versus 26695, 41–45 for Sheeba versus 26695 or J99 and 204–238 for Sheeba versus H. hepaticus [18], W. succinogenes [85] or C. jejuni [86]. (863 KB PDF) [file pgen.0020120.sg006.pdf]

## Slide 1
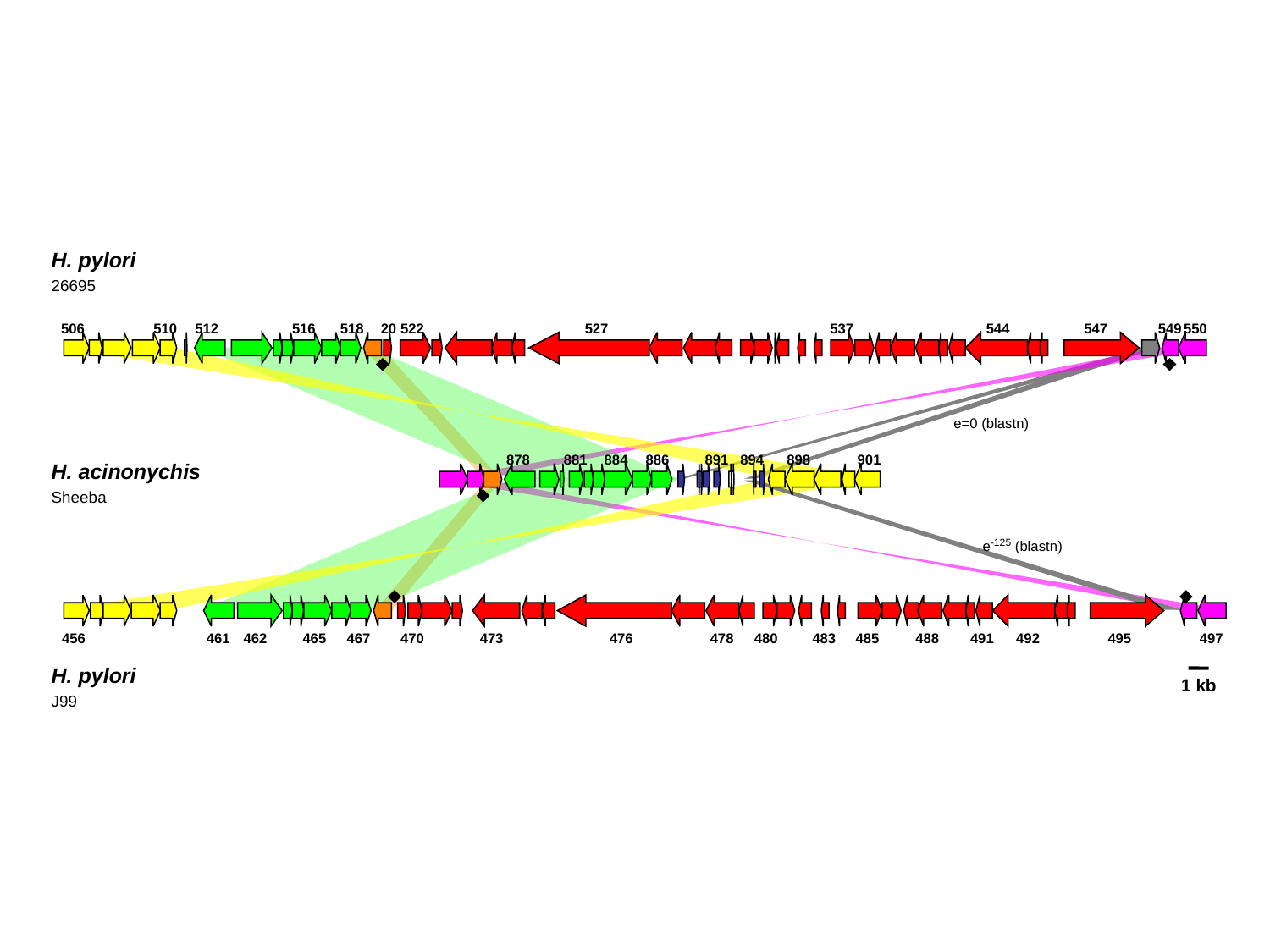

H. pylori
26695
506
510
512
516
518
20
522
527
537
544
547
549
550
e=0 (blastn)
878
881
884
886
891
894
898
901
H. acinonychis
Sheeba
e-125 (blastn)
456
461
462
465
467
470
473
476
478
480
483
485
488
491
492
495
497
H. pylori
J99
1 kb

Supplement: Figure S7 — The cagPAI genes of H. pylori 26695 and J99 (red) are flanked by pairs of 31 bp repeats [87], as indicated by diamonds. The cagPAI region between the repeats is lacking in the Sheeba genome and only one of the repeats is present. The region flanking these repeats is syntenic in all three genomes, as indicated by color coding but has suffered some rearrangements. As part of these rearrangements, the syntenic cluster in yellow has been inverted and transposed downstream of the green cluster and additional sequences have been inserted into the grey region of Sheeba, resulting in a cluster of fragmented genes between Hac0887 and Hac0894, which shows a partial homology to HP0548 in 26695 and the region between jhp0495 and jhp0496 in J99. (74 KB PPT) [file pgen.0020120.sg007.ppt]

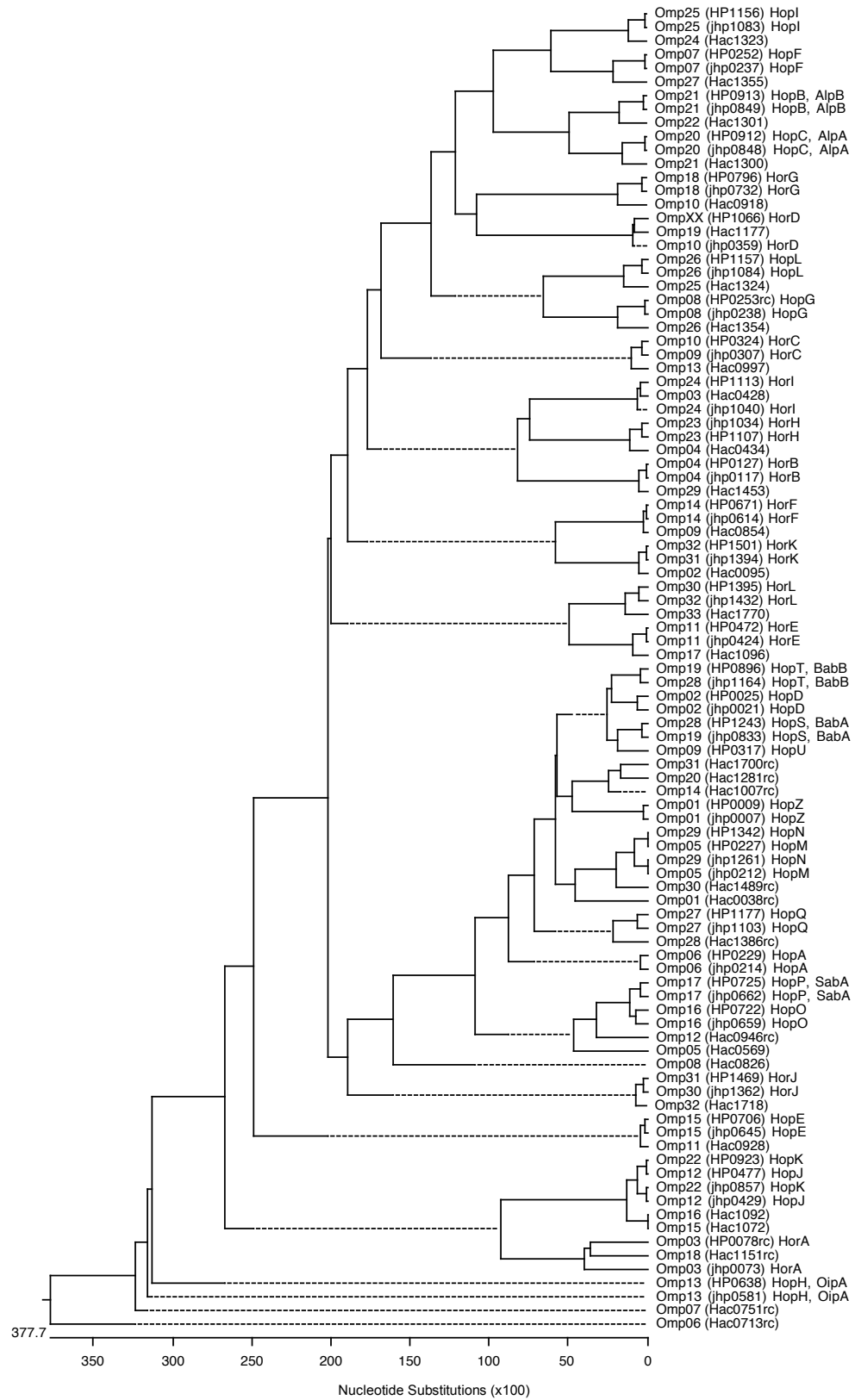

Supplement: Figure S8 — Omps of the Hop- and Hor- families [88] were assigned sequential numbers based on their genomic positions within 26695 (genes labelled HPxxxx) and J99 (genes labelled jhpxxxx) [16,17], except for HP1066 (HorD), which was designated OmpXX. We assigned independent, sequential numbers to OMPs within Sheeba based on their genomic locations within its genome; these numbers differ from those of orthologs in the H. pylori genomes due to genomic rearrangements. Orthologs between OMPs in the three genomes were determined by phylogenetic clustering of predicted proteins. Where pseudo-genes existed due to fragmentation, the clustering was based on reconstructed genes designated Hacxxxxrc for Sheeba and HPxxxxrc (Omp08, Omp03) for 26695. All Sheeba OMP genes have close orthologs within 26695 and/or J99, except for Omps 6 (Hac0713rc), 7 (Hac0751rc), and 8 (Hac0826) and all OMP genes within 26695 or J99 possess orthologs in Sheeba, except for Omps 1 (HP0009), 2 (HP0025), 6 (HP0229), 9 (HP0317), 13 (HP0638), 19 (HP0896), and 28 (HP1243). Of the Sheeba Omps that were reconstructed from gene fragments, seven (Omps 1, 12, 14, 20, 28, 30, 31) are of the Hop family, one (Omp18) is of the Hor family, and two (Omps 6, 7) are distantly related to both families. Dotted lines represent truncated numbers of nucleotide substitutions. (46 KB PDF) [file pgen.0020120.sg008.pdf]
