# Supplementary material for: Who Ate Whom? Adaptive Helicobacter Genomic Changes That Accompanied a Host Jump from Early Humans to Large Felines
Source: PLoS Genet. 2006 Jul 28;2(7):e120. doi: 10.1371/journal.pgen.0020120 (PMC1523251; doi:10.1371/journal.pgen.0020120)
Supplement: Figure S5 — (A and B) and (D–G) show normalized blast scores of individual CDSs between Sheeba and other genomes, sorted by the CDS order within Sheeba, within which features of particular interest are highlighted by colors (see color code at the bottom). Cumulative normalized blast scores, sorted in descending order, are shown in (H). The normalized blast scores relative to H. pylori genomes in (A and B) were high for most CDSs, e.g. the highly conserved F0F1 ATP synthase or ribosomal proteins involved in translation. CDSs in (A and B) with low normalized blast scores are unique to Sheeba, and were attributed to HGT. These include the neuACB-cst cluster, two prophages, plasmid-associated genes and restriction and modification enzymes. Fragmentation within certain genes, e.g., vacA, was associated with a series of CDSs with intermediate normalized blast scores. Note that normalized blast scores against other Campylobacterales (D–F) were lower and against the unrelated Escherichia coli (G) were much lower than in comparisons with H. pylori (A and B). (C) is similar to (A), except that it shows a comparison between two H. pylori genomes and that most of the normalized Blast scores are high. Two arrows in (C) indicate hypothetical proteins flanked by the insertion sequence elements IS605, which correspond to regions 1 (left) and 3 of the five distinct G + C regions in 26695 [16]. (3.7 MB PPT) [file pgen.0020120.sg005.ppt]

## Slide 1
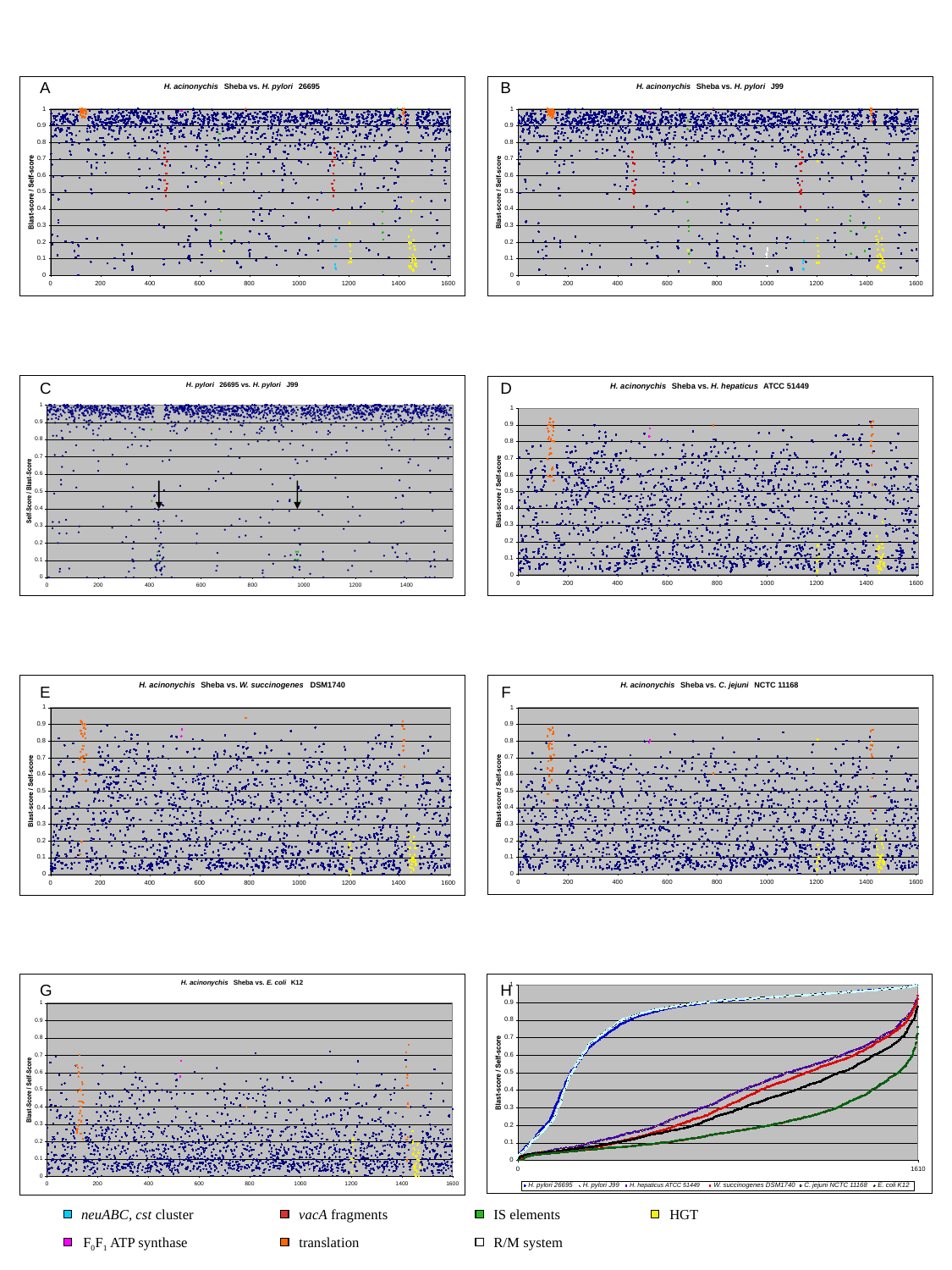

neuABC, cst cluster
F0F1 ATP synthase
vacA fragments
translation
IS elements
R/M system
HGT
A
B
C
D
E
F
G
H
