# Supplementary material for: Who Ate Whom? Adaptive Helicobacter Genomic Changes That Accompanied a Host Jump from Early Humans to Large Felines
Source: PLoS Genet. 2006 Jul 28;2(7):e120. doi: 10.1371/journal.pgen.0020120 (PMC1523251; doi:10.1371/journal.pgen.0020120)
Supplement: Table S1 — (29 KB DOC) [file pgen.0020120.st001.doc]

**Table S1. Synonymous and non-synonymous distances in pairwise comparisons of 612 conserved genes between three *Helicobacter* genomes**.

| Parameter |  | Comparison |  |
| --- | --- | --- | --- |
|  | Sheeba-26695 | Sheeba-J99 | 26695-J99 |
| Synonymous differences | 35,843 | 35,919 | 19,718 |
| Non-synonymous differences | 14,708 | 14,663 | 6,839 |
| D_S_ [19] | 0.3788 | 0.3798 | 0.1846 |
| D_S_ (mod. Nei-Gojobori) | 0.2404 | 0.2410 | 0.1227 |
| D_N_ [19] | 0.0333 | 0.0332 | 0.0153 |
| D_N_ (mod. Nei-Gojobori) | 0.0381 | 0.0380 | 0.0175 |

Number of genes scanned: 612. Number of nucleotides: 571,641. Potential synonymous and non-synonymous sites [19]:120,526; 451,709. R = P/Q (100,459/26,814) = 3.7465. Potential synonymous and non-synonymous site (modified Nei-Gojobori) based on R: 174,273; 395,527.
