# Supplementary material for: Who Ate Whom? Adaptive Helicobacter Genomic Changes That Accompanied a Host Jump from Early Humans to Large Felines
Source: PLoS Genet. 2006 Jul 28;2(7):e120. doi: 10.1371/journal.pgen.0020120 (PMC1523251; doi:10.1371/journal.pgen.0020120)
Supplement: Table S2 — (192 KB PDF) [file pgen.0020120.st002.pdf]

**Table S2**. **Fragmented genes in Sheeba and their orthologs in 26695 and/or J99.**

| *Helicobacter acinonychis* **Sheeba** | | *Helicobacter pylori* **26695** | | *Helicobacter pylori* **J99** | | Functional annotation | |
|---|---|---|---|---|---|---|---|
| **ORF #** | **Length (aa)** | **ORF #** | **Length (aa)** | **ORF #** | **Length (aa)** | **Gene product** | **COG Category Number/Description/ID** |
| **Hac0011**<br>**Hac0012** | 365<br>852 | **HP1517** | 1279 | **jhp1409** | 1252 | type II DNA modification enzyme | COG1002, Type II restriction enzyme methylase subunits, Defense mechanisms, V. |
| **Hac0033**<br>**Hac0034** | 128<br>114 | --- | --- | **jhp1463** | 367 | conserved hypothetical protein | COG1106, Predicted ATPases, R. |
| **Hac0035**<br>**Hac0036**<br>**Hac0037**<br>**Hac0038** | 46<br>165<br>69<br>123 | **HP0009** | 634 | **jhp0007** | 668 | outer membrane protein Omp1 | n.d. |
| **Hac0067**[pg]<br>**Hac0068**[pg] | 365<br>194 | **[HP1417m]** | 556 | **[jhp1312]** | 553 | putative metal-dependent hydrolase | COG2194, Predicted membrane-associated metal-dependent hydrolase, R. |
| **Hac0085**<br>**Hac0087**<br>**Hac0089** | 71<br>35<br>33 | **HP0050** | 232 | **jhp0043** | 230 | type II DNA modification enzyme methhyltransfera DpnA | COG0863, DNA modification methylase, Replication, L. |
| **Hac0091**<br>**Hac0092**<br>**Hac0093**<br>**Hac0094** | 55<br>83<br>101<br>81 | **HP0051** | 355 | --- | --- | DDEM, DNA-methyltransferase | COG0270, Site-specific DNA methylase, Replication, L. |
| **Hac0096** | 479 | **HP1283** | 485 | --- | --- | conserved hypothetical protein | COG0859, ADP-heptose:LPS heptosyltransferase, M. |
| **Hac0128**<br>**Hac0129** | 58<br>119 | --- | --- | --- | --- | conserved hypothetical protein | n.d. |
| **Hac0179** | 144 | --- | --- | **jhp0932** | 156 | conserved hypothetical protein | n.d. |
| **Hac0180**<br>**Hac0181**<br>**Hac0182**<br>**Hac0183** | 55<br>60<br>50<br>68 | **HP1371** | 968 | **jhp1285** | 972 | type III restriction enzyme | n.d. |

| | | | | | | | |
|---|---|---|---|---|---|---|---|
| **Hac0184** | 38 | | | | | | |
| **Hac0185** | 290 | **HP1368** | 287 | --- | --- | modification methylase Mjai | COG0863, DNA modification methylase, L. |
| **Hac0186**<br>**Hac0187** | 90<br>171 | **HP1367** | 260 | --- | --- | type IIS restriction enzyme M1 protein | COG0863, DNA modification methylase, L. |
| **Hac0233**<br>**Hac0234**<br>**Hac0235**<br>**Hac0236**<br>**Hac0237**<br>**Hac0238** | 153<br>153<br>74<br>31<br>90<br>41 | **HP1252** | 594 | **jhp1173** | 595 | ABC-type oligopeptide transport system, periplasmic component fragment 2 | COG4166, ABC-type oligopeptide transport system periplasmic component, E. |
| **Hac0239**<br>**Hac0240**<br>**Hac0241** | 39<br>77<br>197 | **HP1251** | 348 | **jhp1172** | 348 | oligopeptide ABC transporter, permease protein | COG4174, ABC-type uncharacterized transport system permease component, R. |
| **Hac0305** | 172 | **HP1209** | 172 | --- | --- | ulcer-associated gene restriction endonuclease | n.d. |
| **Hac0346** | 424 | **HP0164**<br>**HP0165** | 254<br>173 | **jhp0151** | 442 | two-component sensor histidine kinase | COG0642, Signal transduction histidine kinase, T. |
| **Hac0362** | 447 | --- | --- | **jhp0164** | 448 | type II restriction enzyme | COG1401, GTPase subunit of restriction endonuclease, V. |
| **Hac0363** | 405 | --- | --- | **jhp0165** | 406 | conserved hypothetical protein | n.d. |
| **Hac0372** | 708 | **HP1116** | 957 | **jhp1044** | 1154 | conserved hypothetical protein | n.d. |
| **Hac0373** | 101 | **HP1115** | 228 | **jhp1042** | 267 | conserved hypothetical protein | n.d. |
| **Hac0374** | 89 | **HP0189** | 177 | **jhp0175** | 177 | putative membrane protein | COG2862, Predicted membrane protein, S. |
| **Hac0389**<br>**Hac0390** | 71<br>467 | **HP0030** | 593 | **jhp0026** | 565 | conserved hypothetical protein | n.d. |
| **Hac0392** | 247 | **HP1186** | 202 | **jhp1112** | 247 | carbonic anhydrase | n.d. |
| **Hac0416** | 82 | **HP0390** | 166 | **jhp0991** | 166 | thiol peroxidase | COG2077, Peroxiredoxin, O. |
| **Hac0425** | 96 | **HP1115** | 228 | **jhp1042** | 267 | conserved hypothetical protein | n.d. |
| **Hac0446** | 370 | **HP1116** | 957 | **jhp1044** | 1154 | conserved hypothetical protein | n.d. |
| **Hac0471** | 168 | **HP0384** | 250 | **jhp0997** | 248 | conserved hypothetical protein | n.d. |
| **Hac0477** | 215 | **HP0186** | 404 | **jhp0174** | 567 | conserved hypothetical protein | n.d. |

| | | | | | | | |
|---|---|---|---|---|---|---|---|
| **Hac0478**<br>**Hac0479**<br>**Hac0480** | 41<br>128<br>43 | **HP0187**<br>**HP0188** | 95<br>33 | | | | |
| **Hac0481**<br>**Hac0482**<br>**Hac0483** | 409<br>56<br>82 | **HP0651** | 476 | **jhp0596** | 454 | alpha (1,3)-fucosyltransferase, FucT | n.d. |
| **Hac0487** | 122 | **HP0651** | 476 | **jhp0596** | 454 | alpha (1,3)-fucosyltransferase, FucT', N-terminal fragment | n.d. |
| **Hac0493** | 185 | **HP0368** | 133 | **jhp1013** | 182 | conserved hypothetical protein | n.d. |
| **Hac0494** | 320 | **HP0369**<br>**HP0369$^{m}$** | 236<br>58 | **jhp1012** | 320 | putative type II DNA modification enzyme (methyltransferase) | n.d. |
| **Hac0498**<br>**Hac0499** | 139<br>177 | **---** | --- | **---** | --- | hypothetical protein | n.d. |
| **Hac0502**<br>**Hac0503**<br>**Hac0504**<br>**Hac0505**<br>**Hac0506**<br>**Hac0507**<br>**Hac0508**<br>**Hac0510**<br>**Hac0511**<br>**Hac0512**<br>**Hac0513**<br>**Hac0514**<br>**Hac0515** | 72<br>44<br>66<br>90<br>40<br>106<br>176<br>92<br>71<br>38<br>70<br>96<br>64 | **HP0887** | 1290 | **jhp0819** | 1288 | vacuolating cytotoxin VacA' | pfam03797, Autotransporter beta-domain. |
| **Hac0519** | 240 | **HP0262** | 200 | **jhp0246**<br>**jhp0247** | 172<br>69 | Putative type II restriction enzyme MjaVIP | n.d. |
| **Hac0586** | 260 | **HP1127**<br>**HP1128** | 193<br>84 | **jhp1056** | 263 | conserved hypothetical protein | n.d. |
| **Hac0611** | 299 | **HP0423** | 308 | **jhp0958**<br>**jhp0959** | 51<br>218 | conserved hypothetical protein | n.d. |
| **Hac0613**<br>**Hac0614** | 59<br>118 | **---** | --- | **jhp0957** | 200 | conserved hypothetical protein | n.d. |

| | | | | | | | |
|---|---|---|---|---|---|---|---|
| **Hac0615** | 140 | --- | --- | **jhp0956** | 161 | conserved hypothetical protein | n.d. |
| **Hac0616** | 241 | --- | --- | **jhp0955** | 217 | conserved hypothetical protein | n.d. |
| **Hac0617**<br>**Hac0618** | 41<br>107 | --- | --- | **jhp0954** | 146 | conserved hypothetical protein | n.d. |
| **Hac0626**<br>**Hac0627** | 144<br>421 | **HP1409** | 578 | **jhp1301**<br>**jhp1302** | 314<br>201 | conserved hypothetical protein | COG1479, Uncharacterized conserved protein, Function Unknown, S. |
| **Hac0628**<br>**Hac0629**<br>**Hac0630** | 39<br>112<br>147 | --- | --- | **jhp0914** | 411 | conserved hypothetical protein | n.d. |
| **Hac0647**<br>**Hac0648** | 35<br>113 | **HP0766** | 274 | **jhp0704** | 182 | conserved hypothetical protein | n.d. |
| **Hac0649**<br>**Hac0650**<br>**Hac0651**<br>**Hac0652** | 38<br>61<br>39<br>106 | **HP0764**<br>**HP0765** | 428<br>102 | **jhp0701**<br>**jhp0702**<br>**jhp0703** | 117<br>168<br>286 | hypothetical protein | COG0727, Predicted Fe-S-cluster oxidoreductase, R. |
| **Hac0686**<br>**Hac0687**<br>**Hac0688**<br>**Hac0689** | 90<br>108<br>58<br>75 | **HP0733**<br>**HP0732** | 521<br>118 | **jhp0670**<br>**jhp0669** | 192<br>371 | hypothetical protein | n.d. |
| **Hac0690**<br>**Hac0691**<br>**Hac0692**<br>**Hac0693** | 59<br>216<br>148<br>68 | **HP0731** | 573 | **jhp0668** | 569 | conserved hypothetical protein | n.d. |
| **Hac0702** | 814 | **HP0915**<br>**HP0916** | 562<br>249 | **jhp0851** | 815 | iron-regulated outer membrane protein FrpB | COG1629, CirA, Outer membrane receptor proteins, mostly Fe transport, Inorganic ion transport and metabolism, P. |
| **Hac0710**<br>**Hac0711** | 213<br>186 | --- | --- | **---** | --- | type III methylase | COG2189, Adenine specific DNA methylase Mod, DNA replication, recombination, and repair, L. |
| **Hac0713**<br>**Hac0714** | 136<br>35 | **HP0638** | 305 | **jhp0581** | 307 | outer membrane protein Omp6 | n.d. |
| **Hac0731** | 278 | **HP0629** | 681 | **jhp0572** | 683 | conserved hypothetical protein | COG1479, Uncharacterized |

| | | | | | | | |
|---|---|---|---|---|---|---|---|
| **Hac0732** | 415 | | | | | | conserved protein, Function Unknown, S. |
| **Hac0733** | 347 | **HP0627**<br>**HP0628** | 110<br>225 | **jhp0571** | 352 | conserved hypothetical protein | COG0790, FOG: TPR repeat SEL1 subfamily, R. |
| **Hac0751**<br>**Hac0752** | 119<br>170 | **HP0638** | 305 | **jhp0581** | 307 | outer membrane protein Omp7 | n.d. |
| **Hac0753**<br><br><br><br>**Hac0755**<br>**Hac0756** | 134<br><br><br><br>139<br>279 | **HP1008**<br><br><br><br>**HP0413** | 138<br><br><br><br>440 | **jhp0827**<br><br><br><br>**jhp0826** | 64<br><br><br><br>422 | ISHa1942 transposase A (IS606-like IS element)<br><br><br>ISHa1942 transposase B | COG1943, Transposase and inactivated derivatives, DNA replication, recombination, and repair, L.<br>COG0675, Transposase and inactivated derivatives, DNA replication, recombination, and repair, L. |
| **Hac0758**<br>**Hac0760**<br>**Hac0761**<br><br>**Hac0762** | 32<br>41<br>37<br><br>134 | **---**<br>**---**<br>**---**<br><br>**---** | ---<br>---<br>---<br><br>--- | **---**<br>**---**<br>**---**<br><br>**---** | ---<br>---<br>---<br><br>--- | ISHa1152 transposase B (ISHp608-like IS element)<br><br><br><br>ISHa1152 transposase A, N-terminal fragment | COG0675, Transposase and inactivated derivatives, DNA replication, recombination, and repair, L.<br>COG1943, Transposase and inactivated derivatives, DNA replication, recombination, and repair, L. |
| **Hac0773** | 140 | **HP0719**<br>**HP0720** | 109<br>53 | **jhp0657** | 140 | conserved hypothetical protein | n.d. |
| **Hac0802**<br>**Hac0803** | 264<br>200 | **HP1083** | 479 | **jhp0342** | 479 | outer membrane protein HofB | n.d. |
| **Hac0818**<br>**Hac0819** | 287<br>150 | **HP0498** | 442 | **jhp0450** | 442 | sodium- and chloride-dependent transporter | COG0733, Na+-dependent transporters of the SNF family, R. |
| **Hac0823** | 330 | **HP0502**<br>**HP0503** | 54<br>246 | **jhp0454** | 343 | conserved hypothetical protein | n.d. |
| **Hac0824**<br>**Hac0825** | 44<br>76 | **HP0504**<br>**HP0505** | 49<br>154 | **jhp0455** | 251 | conserved hypothetical protein | n.d. |
| **Hac0861** | 314 | **HP0678**<br>**HP0679** | 36<br>289 | **jhp0620** | 315 | lipopolysaccharide biosynthesis protein, WbpB | COG0673 Predicted dehydrogenases and related |

| | | | | | | | |
|---|---|---|---|---|---|---|---|
| | | | | | | | proteins, R. |
| **Hac0864** | 248 | **HP0684**<br>**HP0685** | 105<br>153 | **jhp0625** | 248 | flagellar biosynthesis protein | COG1338, Flagellar biosynthesis pathway component FliP, NU. |
| **Hac0867** | 270 | **HP0688**<br>**HP0689** | 166<br>89 | **jhp0628** | 276 | conserved hypothetical protein | COG3298, Predicted 3-5 exonuclease related to the exonuclease domain of PolB, L. |
| **Hac0868**<br>**Hac0869** | 42<br>47 | **HP0556** | 145 | **jhp0503** | 145 | hypothetical protein | n.d. |
| **Hac0879**<br>**Hac0880**<br>**Hac0881** | 301<br>69<br>226 | **HP0513** | 658 | **jhp0462** | 706 | conserved hypothetical protein | COG1479, Uncharacterized conserved protein, Function unknown, S. |
| **Hac0910**<br>**Hac0911**<br>**Hac0912** | 162<br>31<br>30 | **HP0790** | 431 | **jhp0726** | 454 | restriction enzyme HsdS | COG0732, HsdS Restriction endonuclease S subunits, V. |
| **Hac0934**<br>**Hac0935**<br>**Hac0936** | 32<br>124<br>31 | **[HP0712]**<br>**[HP0713]** | 124<br>114 | **[jhp0651]** | 234 | conserved hypothetical protein[p] | COG3177, Uncharacterized conserved protein, S. |
| **Hac0942**<br>**Hac0943**<br>**Hac0944**<br>**Hac0945**<br>**Hac0946** | 84<br>44<br>69<br>49<br>164 | **HP0725** | 629 | **[jhp0659, contingency off ]** | 638 | outer membrane protein Omp12 | n.d. |
| **Hac0953** | 332 | **HP0694** | 257 | **jhp0634** | 336 | putative outer membrane protein | COG3528, Uncharacterized protein conserved in bacteria, S. |
| **Hac0976** | 403 | **HP0345**<br>**HP0346** | 111<br>260 | --- | --- | conserved hypothetical protein | n.d. |
| **Hac0977** | 261 | **HP0343**<br>**HP0344** | 140<br>154 | --- | --- | conserved hypothetical protein | n.d. |
| **Hac0978**<br>**Hac0980** | 80<br>54 | **HP0342** | 130 | --- | --- | hypothetical protein | n.d. |
| **Hac0994**<br>**Hac0995** | 283<br>229 | **HP0326** | 517 | **[jhp0309, pseudogene, frameshift]** | 516 | NeuA; CMP-N-acetylneuraminic acid synthetase | COG1083, NeuA, CMP-N-acetylneuraminic acid synthetase, Cell envelope biogenesis, outer membrane, M (N-terminal); |

| | | | | | | | |
|---|---|---|---|---|---|---|---|
| | | | | | | | COG3980, Spore coat polysaccharide biosynthesis protein predicted glycosyltransferase, M (C-terminal). |
| **Hac1007**<br>**Hac1010** | 145<br>76 | **HP0317** | 745 | **---** | --- | outer membrane protein Omp14 | n.d. |
| **Hac1012**<br>**Hac1013** | 194<br>184 | **HP0313** | 381 | **jhp0298** | 387 | nitrite extrusion protein NarK | COG2814, AraJ, Arabinose efflux permease, Carbohydrate transport and metabolism, G. |
| **Hac1038**<br>**Hac1039**<br>**Hac1040** | 101<br>171<br>157 | **HP0963** | 447 | **jhp0897** | 226 | conserved hypothetical protein | n.d. |
| **Hac1041**<br>**Hac1042**<br>**Hac1043**<br>**Hac1044**<br>**Hac1045**<br>**Hac1046**<br>**Hac1047** | 97<br>65<br>80<br>169<br>100<br>38<br>143 | **HP0964**<br>**HP0965** | 363<br>443 | **jhp0898**<br>**jhp0899** | 244<br>500 | conserved hypothetical protein | n.d. |
| **Hac1048**<br>**Hac1049**<br>**Hac1050**<br>**Hac1051** | 45<br>30<br>185<br>34 | **HP0966** | 549 | **jhp0900**<br>**jhp0901** | 340<br>166 | conserved hypothetical protein | n.d. |
| **Hac1052** | 78 | **HP0967** | 95 | **---** | --- | virulence-associated protein VapD | COG3309, Uncharacterized virulence-associated protein D, S. |
| **Hac1053** | 93 | **---** | --- | **jhp0902** | 93 | conserved hypothetical protein | n.d. |
| **Hac1080** | 154 | **---** | --- | **jhp0870** | 668 | outer membrane protein HomB, N-terminal fragment | n.d. |
| **Hac1084** | 219 | **HP0931**<br>**HP0932** | 146<br>100 | **jhp0866** | 219 | conserved hypothetical protein | n.d. |
| **Hac1091** | 38 | **HP0924** | 68 | **jhp0858** | 68 | tautomerase | COG1942, Uncharacterized protein 4-oxalocrotonate tautomerase homolog, R. |
| **Hac1094** | 108 | **HP0488** | 957 | **jhp0440** | 912 | conserved hypothetical protein | n.d. |

| | | | | | | | |
|---|---|---|---|---|---|---|---|
| **Hac1095** | 227 | | | | | | |
| **Hac1107**<br>**Hac1108**<br>**Hac1109**<br>**Hac1110** | 112<br>104<br>456<br>309 | **[HP0464, contingency]** | 1055 | **[jhp0416, frameshift]** | --- | HsdR; type I restriction enzyme R | COG0610, Type I site-specific restriction-modification system, Restriction subunit and related helicases, Defense mechanisms,V. |
| **Hac1123** | 476 | **HP1018**<br>**HP1019** | 48<br>443 | **jhp0405** | 476 | serine protease HtrA | COG0265, DegQ, Trypsin-like serine proteases, typically periplasmic, contain C-terminal PDZ domain, O. |
| **Hac1148**<br>**Hac1149**<br>**Hac1150**<br>**Hac1151**<br>**Hac1152** | 91<br>68<br>122<br>64<br>63 | **HP0079**<br>**HP0078** | 595<br>85 | **jhp0073** | 255 | outer membrane protein Omp18 | n.d. |
| **Hac1156** | 662 | **HP1045** | 662 | --- | --- | acetyl-coenzyme A synthetase, AcoE | COG0365, Acyl-coenzyme A synthetases/AMP-(fatty) acid ligases, Lipid transport and metabolism, I. |
| **Hac1185**<br>**Hac1186**<br>**Hac1187** | 103<br>93<br>30 | **HP1074** | 262 | **jhp0351** | 272 | conserved hypothetical protein | n.d. |
| **Hac1207** | 201 | **HP0836**<br>**HP0837** | 119<br>102 | **jhp0775** | 201 | conserved hypothetical protein with DUF400 domains | n.d. |
| **Hac1218** | 499 | **HP0855** | 527 | **---** | --- | alginate o-acetyltransferase, AlgI | COG1696, DltB, Predicted membrane protein involved in D-alanine export, Cell envelope biogenesis, outer membrane, M. |
| **Hac1219** | 365 | **HP856** | 364 | **---** | -- | conserved hypothetical protein | n.d. |
| **Hac1244**<br>**Hac1245**<br>**Hac1246**<br>**Hac1247** | 138<br>308<br>44<br>107 | **HP0373** | 700 | **jhp1008** | 751 | outer membrane protein HomC | n.d. |
| **Hac1253**<br>**Hac1254** | 64<br>96 | **HP0887** | 1290 | **jhp0819** | 1288 | vacuolating cytotoxin VacA | pfam03797, Autotransporter beta-domain. |

| | | | | | | | |
|---|---|---|---|---|---|---|---|
| **Hac1255**<br>**Hac1256**<br>**Hac1257**<br>**Hac1258**<br>**Hac1260**<br>**Hac1261**<br>**Hac1262**<br>**Hac1263**<br>**Hac1264**<br>**Hac1265**<br>**Hac1266** | 70<br>38<br>71<br>92<br>176<br>106<br>40<br>99<br>66<br>44<br>72 | | | | | | |
| **Hac1278**<br>**Hac1281** | 76<br>145 | **HP0317** | 745 | **jhp0833** | 744 | outer membrane protein Omp20 | n.d. |
| **Hac1290** | 507 | **HP0905** | 223 | **jhp0841** | 519 | phosphate acetyltransferase Pta | COG0280, Phosphotransacetylase, Energy production and conversion, C. |
| **Hac1332**<br>**Hac1333**<br>**Hac1344**<br>**Hac1345** | 78<br>107<br>124<br>52 | **HP1165** | 386 | **jhp1092** | 386 | major facilitator superfamily permease | n.d. |
| **Hac1354** | 471 | **HP0253**<br>**HP0254** | 38<br>231 | **jhp0238** | 471 | outer membrane protein Omp23 | n.d. |
| **Hac1357**<br>**Hac1358**<br>**Hac1359**<br>**Hac1360**<br>**Hac1361** | 46<br>81<br>62<br>75<br>53 | **HP0251** | 338 | **jhp0236** | 338 | ABC-type oligopeptide transport, permease component OppC | COG4239, ABC-type uncharacterized transport system permease component, R. |
| **Hac1362**<br>**Hac1363**<br>**Hac1365**<br>**Hac1366** | 114<br>98<br>106<br>119 | **HP0250** | 516 | **jhp0235** | 516 | ABC-type oligopeptide transport, ATP-binding component OppD | COG1123, ATPase components of various ABC-type transport systems contain duplicated ATPase, R |
| **Hac1386**<br>**Hac1387**<br>**Hac1388** | 181<br>119<br>130 | **HP1177** | 641 | **jhp1103** | 643 | outer membrane protein Omp28 | n.d. |

| | | | | | | | |
|---|---|---|---|---|---|---|---|
| **Hac1389** | 84 | | | | | | |
| **Hac1392** | 202 | **HP0611**<br>**HP0612** | 166<br>79 | **jhp0299** | 251 | conserved hypothetical protein | n.d. |
| **Hac1393** | 3216 | **HP0609**<br>**HP0610** | 1238<br>1943 | **jhp0556** | 3194 | vacuolating cytotoxin paralog VacA | COG5651, PPE-repeat proteins, Cell motility and secretion, N. |
| **Hac1404**<br>**Hac1405**<br>**Hac1406**<br>**Hac1407** | 86<br>33<br>163<br>47 | **HP0600** | 593 | **jhp0547** | 589 | putative secretion/efflux ABC transporter, ATP-binding protein | COG1132, MdlB, ABC-type multidrug/protein/lipid transport system, ATPase component, Q. |
| **Hac1426** | 76 | --- | --- | **jhp0533** | 78 | hypothetical protein | n.d. |
| **Hac1445**<br>**Hac1446** | 85<br>148 | **HP0568** | 255 | **jhp0515** | 255 | conserved hypothetical protein | n.d. |
| **Hac1466** | 54 | **HP0502**<br>**HP0503** | 54<br>246 | **jhp0454** | 343 | conserved hypothetical protein | n.d. |
| **Hac1483**<br>**Hac1485**<br>**Hac1486** | 32<br>41<br>37 | ---<br>---<br>--- | ---<br>---<br>--- | ---<br>---<br>--- | ---<br>---<br>--- | ISHa1152 transposase B (ISHp608-like IS element) | COG0675, Transposase and inactivated derivatives, DNA replication, recombination, and repair, L. |
| **Hac1487** | 134[2] | --- | --- | --- | --- | ISHa1152 transposase A, N-terminal fragment | COG1943, Transposase and inactivated derivatives, DNA replication, recombination, and repair, L. |
| **Hac1489**<br>**Hac1490**<br>**Hac1491** | 216<br>149<br>254 | **HP0227** | 691 | **jhp0212** | 696 | outer membrane protein Omp30 | n.d. |
| **Hac1502**<br>**Hac1503** | 246<br>132 | **HP0216** | 368 | **jhp0202** | 368 | Dxr, 1-deoxy-D-xylulose 5-phosphate reductoisomerase | pfam02670, DXP_reductoisom, 1-deoxy-D-xylulose 5-phosphate reductoisomerase. |
| **Hac1510**<br>**Hac1511** | 154<br>43 | **HP0093**<br>**HP0094** | 146<br>155 | **[jhp0086 artificial, Slipped-strand]** | 299 | alpha-1,2-fucosyltransferase FucT | pfam01531, Glycosyl transferase family 11. |
| **Hac1513**<br>**Hac1514** | 230<br>113 | - | - | - | - | HrgA | COG2958, Uncharacterized protein conserved in bacteria, S. |

| | | | | | | | |
|---|---|---|---|---|---|---|---|
| **Hac1539**<br>**Hac1540**<br>**Hac1541**<br>**Hac1543**<br>**Hac1544** | 94<br>98<br>131<br>68<br>64 | **HP0066** | 831 | **jhp0061** | 806 | conserved hypothetical protein with COG1674 domain | COG1674, FtsK DNA segregation ATPase FtsK/SpoIIIE and related proteins, Cell division and chromosome partitioning, D. |
| **Hac1547**<br>**Hac1548**<br>**Hac1549** | 43<br>87<br>42 | **HP0063** | 496 | **jhp0058** | 500 | conserved hypothetical protein | n.d. |
| **Hac1551** | 37 | **HP1096**<br>**(=HP1535)** | 142 | **---** | --- | ISHa1675 transposase A, N-terminal fragment<br>(IS605-like IS element) | COG1943, Transposase and inactivated derivatives, DNA replication, recombination, and repair, L. |
| **Hac1552**<br>**Hac1553**<br>**Hac1554** | 47<br>68<br>41 | **HP0061** | 189 | **jhp0056** | 189 | hypothetical protein | n.d. |
| **Hac1555** | 51 | **HP0060** | 813 | **jhp0053**<br>**jhp0054**<br>**jhp0055** | 143<br>522<br>78 | hypothetical protein | n.d. |
| **Hac1562**<br>**Hac1563** | 730<br>296 | **HP0205**<br>**HP0206** | 793<br>108 | **jhp0191**<br>**jhp0192** | 916<br>85 | conserved hypothetical protein | n.d. |
| **Hac1569**<br>**Hac1570**<br>**Hac1571** | 123<br>65<br>105 | **HP1193** | 329 | **---** | --- | aldo-keto reductase Tas1 | COG0667, Tas, Predicted oxidoreductases, Energy production and conversion, C. |
| **Hac1593** | 753 | **HP1215**<br>**HP1216** | 80<br>660 | **jhp1138** | 766 | organic solvent tolerance protein OstA | COG1452, Organic solvent tolerance protein OstA, Cell wall, M. |
| **Hac1655**<br>**Hac1656** | 357<br>450 | **HP1433** | 856 | **jhp1326** | 792 | conserved hypothetical protein | n.d. |
| **Hac1661** | 69 | **HP1438** | 338 | **jhp1331**<br>**e-19** | 161 | conserved hypothetical protein, N-terminal fragment | n.d. |
| **Hac1700**<br>**Hac1705** | 145<br>113 | **HP0317** | 745 | **jhp0007** | 668 | outer membrane protein Omp34 | n.d. |

| | | | | | | | |
|---|---|---|---|---|---|---|---|
| **Hac1706** | 36 | | | | | | |
| **Hac1712** | 218 | **HP1000** | 218 | **jhp0935** | 180 | chromosome partitioning protein ParA | COG1192, ATPases involved in chromosome partitioning, Cell cycle control, D. |
| **Hac1713** | 94 | **HP1001** | 94 | **---** | --- | conserved hypothetical protein | n.d. |
| **Hac1714** | 392 | **HP1002** | 394 | **---** | --- | conserved hypothetical protein | n.d. |
| **Hac1715** | 107 | **HP1003** | 370 | **---** | --- | conserved hypothetical protein, C-terminal fragment | n.d. |
| **Hac1720** | 774 | **---** | --- | **jhp1297** | 779 | type III restriction-modification | pfam04851, ResIII, Type III restriction enzyme, res subunit. (EC:3.1.21.5). |
| **Hac1721** | 642 | **HP1369m** | 809 | **jhp1296** | 620 | type III restriction-modification system: methyltransferase Mod | COG2189, Adenine specific DNA methylase Mod, DNA replication, recombination, and repair, L. |
| **Hac1762**<br>**Hac1763**<br>**Hac1764** | 258<br>147<br>44 | **HP1017** | 519 | **jhp0406** | 475 | amino acid permease RocE' | COG0833, LysP, Amino acid transporters ,Amino acid transport and metabolism, E. |
| **Hac1766**<br>**Hac1767** | 39<br>55 | **HP1397** | 271 | **jhp1430** | 282 | conserved hypothetical protein | COG1479, Uncharacterized conserved protein, Function Unknown, S. |
| **Hac1768**<br>**Hac1769** | 67<br>59 | **HP1396** | 288 | **jhp1431** | 293 | conserved hypothetical protein | COG1479, Uncharacterized conserved protein, Function Unknown, S. |
| **Hac1783** | 218 | **HP1537** | 228 | **---** | --- | conserved hypothetical protein | n.d. |

The cluster of orthologous groups (COG) category and respective gene names is listed even if not detectable in all fragmented open reading frames. The *H. acinonychis* Sheeba specific omps numbered starting with *omp*1 downstream of *dnaA* at the origin of replication, ' duplicated and fragmented in *H. acinonychis* Sheeba, [m]modified gene prediction after comparative analysis of all *Helicobacter* genomes, n.d., no putative conserved domains have been detected.
