# Supplementary material for: Who Ate Whom? Adaptive Helicobacter Genomic Changes That Accompanied a Host Jump from Early Humans to Large Felines
Source: PLoS Genet. 2006 Jul 28;2(7):e120. doi: 10.1371/journal.pgen.0020120 (PMC1523251; doi:10.1371/journal.pgen.0020120)
Supplement: Table S3 — (67 KB PDF) [file pgen.0020120.st003.pdf]

Table S3. Sequence differences of ten fragmented genes between Sheeba and three strains of *H. acinonychis*.

Sheeba		t1	HA5141	Bombay A
**Gene**	**Size (bp)**	**Polymorphic variations relative to Sheeba (number of bp changed)**		
**omp1**<br>(Hac0035-38)	1940	1840<br>0 SNPs	1844<br>0 SNPs<br>1 deletion (1)	960<br>31 SNPs<br>4 insertions (7)<br>2 deletions (3)
**hypothetical protein 1**<br>(Hac0690-3)	2621	2143<br>5 SNPs	1475<br>1 SNP	2566<br>181 SNPs<br>6 insertions (36)<br>3 deletions (3)
**hypothetical protein 2**<br>(Hac0731-2)	2463	2152<br>26 SNPs<br>1 deletion (3)	2412<br>0 SNPs	2377<br>156 SNPs<br>4 insertions (11)<br>4 deletions (15)
**omp12**<br>(Hac0942-6)	2095	2018<br>17 SNPs<br>1 insertion (6)	985<br>2 SNPs	1992<br>81 SNPs<br>3 insertions (3)<br>10 deletions (16)
**omp14**<br>(Hac1007-10)	1425	1361<br>0 SNPs<br>1 insertion (1)<br>1 deletion (1)	1344<br>1 SNP	1572<br>90 SNPs<br>5 insertions (235)<br>3 deletions (6)
**homB**<br>(Hac1244-7)	2193	1209<br>4 SNPs<br>1 deletion (1)	1207<br>0 SNPs	1156<br>58 SNPs<br>5insertions (28)<br>4 deletions (74)
**omp20**<br>(Hac1278-81)	2005	1928<br>0 SNPs<br>2 insertions (12)<br>1 deletion (1)	883<br>0 SNPs<br>1 insertion (1)	Not amplified
**omp28**<br>(Hac1386-9)	2451	2371<br>3 SNPs	2368<br>0 SNPs	1790<br>59 SNPs<br>4insertions (107)<br>5 deletions (59)
**omp30**<br>(Hac1489-91)	2270	Not amplified	2270<br>1 SNP<br>1 insertion (1)<br>1 deletion (1)	1134<br>59 SNPs<br>2 insertions (3)<br>3 deletions (3)
**vacA**<br>(Hac1253-66)	3815	1110<br>3 SNPs	1110<br>0 SNPs	931<br>52 SNPs<br>3 insertions (3)<br>5 deletions (214)

The data summarizes the length of gene that was sequenced, the number of single nucleotide polymorphisms (SNPs) and any insertions and deletions.
