# Supplementary material for: Who Ate Whom? Adaptive Helicobacter Genomic Changes That Accompanied a Host Jump from Early Humans to Large Felines
Source: PLoS Genet. 2006 Jul 28;2(7):e120. doi: 10.1371/journal.pgen.0020120 (PMC1523251; doi:10.1371/journal.pgen.0020120)
Supplement: Table S4 — (136 KB PDF) [file pgen.0020120.st004.pdf]

Table S4. Unique genes in Sheeba.

Helicobacter acinonychis Sheeba		*Helicobacter pylori* 26695		*Helicobacter pylori* J99		Functional annotation	
**ORF #**	**Length (aa)**	**ORF #**	**Length (aa)**	**ORF #**	**Length (aa)**	**Gene product**	**COG Category Number/Description/ID**
**Hac0067**[pg]<br>**Hac0068**[pg]	365<br>194	**[HP1417m]**	556	**[jhp1312]**	553	putative metal-dependent hydrolase	COG2194, Predicted membrane-associated metal-dependent hydrolase, R.
**Hac0101**	60	---	---	---	---	restriction enzyme HsdS3a[R/M]	COG0732, Restriction endonuclease S subunits, V.
**Hac0111**	82	---	---	---	---	hypothetical protein	n.d.
**Hac0118**	121	---	---	---	---	hypothetical protein	n.d.
**Hac0127**	52	---	---	---	---	hypothetical protein	n.d.
**Hac0128**<br>**Hac0129**	58<br>119	---	---	---	---	conserved hypothetical protein	n.d.
**Hac0130**	53	---	---	---	---	hypothetical protein	n.d.
**Hac0208**[pg]	397	**[HP1283]**	485	---	---	conserved hypothetical protein	COG0859, RfaF, ADP-heptose:LPS heptosyltransferase, Cell envelope biogenesis, outer membrane, M.
**Hac0253**	176	---	---	---	---	hypothetical protein	n.d.
**Hac0283**	418	---	---	---	---	Cytosine-specific DNA methyltransferase (DDEM)[R/M]	COG0270, Dcm, Site-specific DNA methylase, L.
**Hac0284**	366	---	---	---	---	conserved hypothetical protein	n.d.
**Hac0322**	96	---	---	---	---	hypothetical protein	n.d.
**Hac0360**	377	---	---	---	---	Cytosine-specific methyltransferase[R/M]	COG0270, Dcm, Site-specific DNA methylase, L.
**Hac0361**	256	---	---	---	---	conserved hypothetical protein[pp]	n.d.
**Hac0487**	122	**[HP0651]**	476	**[jhp0596]**	454	alpha (1,3)-fucosyltransferase, FucT'	n.d.
**Hac0498**<br>**Hac0499**	139<br>177	---	---	---	---	conserved hypothetical protein	n.d.

**Hac0500**	56	---	---	---	---	hypothetical protein	n.d.
**Hac0502**<br>**Hac0503**<br>**Hac0504**<br>**Hac0505**<br>**Hac0506**<br>**Hac0507**<br>**Hac0508**<br>**Hac0510**<br>**Hac0511**<br>**Hac0512**<br>**Hac0513**<br>**Hac0514**<br>**Hac0515**	72<br>44<br>66<br>90<br>40<br>106<br>176<br>92<br>71<br>38<br>70<br>96<br>64	**[HP0887]**	1290	**[jhp0819]**	1288	vacuolating cytotoxin VacA'	pfam03797, Autotransporter beta-domain.
**Hac0567**	64	---	---	---	---	hypothetical protein	n.d.
**Hac0591**	68	---	---	---	---	hypothetical protein	n.d.
**Hac0620**	108	---	---	---	---	hypothetical protein	n.d.
**Hac0621**	62	---	---	---	---	hypothetical protein	n.d.
**Hac0622**	59	---	---	---	---	hypothetical protein	n.d.
**Hac0623**	142	---	---	---	---	hypothetical protein	n.d.
**Hac0707**	59	---	---	---	---	hypothetical protein	n.d.
**Hac0709**	53	---	---	---	---	hypothetical protein	n.d.
**Hac0710**<br>**Hac0711**	213<br>186	---	---	---	---	type III restriction-modification system methylase[R/M]	COG2189, Adenine-specific DNA methylase Mod, DNA replication, recombination, and repair, L.
**Hac0712**	673	---	---	---	---	conserved hypothetical protein[HGT]	COG1061, SSL2, DNA or RNA helicases of superfamily II, Transcription / DNA replication, recombination, and repair, L.
**Hac0757**	83	---	---	---	---	replication initiation protein RepA, N-terminal fragment[p]	n.d.
**Hac0765**	387	---	---	---	----	MccC-like protein[p]	n.d.
**Hac0766**	167	---	---	---	---	MccB-like protein[p], C-terminal fragment	n.d.

**Hac0776**	71	---	---	---	---	hypothetical protein	n.d.
**Hac0814**	54	---	---	---	---	hypothetical protein	n.d.
**Hac0837**	193	---	---	---	---	hypothetical protein	n.d.
**Hac0852**	322	---	---	---	---	conserved hypothetical protein	n.d.
**Hac0853**	255	---	---	---	---	hypothetical protein	n.d.
**Hac0857**	56	---	---	---	---	hypothetical protein	n.d.
**Hac0887**	95	---	---	---	---	hypothetical protein	n.d.
**Hac0889**	58	---	---	---	---	hypothetical protein	n.d.
**Hac0890**	98	---	---	---	---	hypothetical protein	n.d.
**Hac0891**	99	---	---	---	---	hypothetical protein	n.d.
**Hac0894**	41	---	---	---	---	hypothetical protein	n.d.
**Hac0895**	71	---	---	---	---	hypothetical protein	n.d.
**Hac0921**	142	---	---	---	---	hypothetical protein	n.d.
**Hac0922**	148	---	---	---	---	hypothetical protein	n.d.
**Hac0965**	119	---	---	---	---	hypothetical protein	n.d.
**Hac0966**	177	---	---	---	---	Cytosine-specific DNA methylase[R/M; pp]	COG0270, Dcm, Site-specific DNA methylase, DNA replication, recombination, and repair, L.
**Hac1016**	374	---	---	---	---	type II DNA modification methylase[R/M]	COG0863, DNA modification methylase, DNA replication, recombination, and repair, L.
**Hac1017**	230	---	---	---	---	type II restriction endonuclease[R/M]	n.d.
**Hac1103**	105	---	---	---	---	hypothetical protein	n.d.
**Hac1112**	85	---	---	---	---	hypothetical protein	n.d.
**Hac1192**	76	---	---	---	---	hypothetical protein	n.d.
**Hac1193**	65	---	---	---	---	hypothetical protein	n.d
**Hac1199**	58	---	---	---	---	hypothetical protein	n.d.
**Hac1213**	327	---	---	---	---	site-specific DNA methyltransferase[R/M]	COG0270, Dcm, Site-specific DNA methylase, Replication, L.
**Hac1214**	317	---	---	---	---	type II restriction endonuclease[R/M]	n.d.
**Hac1267**	395	---	---	---	---	putative bifunctional alpha-2,3/-2,8-sialyltransferase (Cst)	pfam06002, CST-I, Alpha-2,3-sialyltransferase (CST-I).

**Hac1268**	422	---	---	---	---	putative bifunctional alpha-2,3/8-sialyltransferase (Cst)	pfam06002, CST-I, Alpha-2,3-sialyltransferase (CST-I). pfam02512,Virulence determinant. The UK protein is an African swine fever virus (ASFV) protein that is highly conserved amongst strains, and is an important viral virulence determinant for domestic pigs.
**Hac1269**	219	---	---	---	---	CMP-sialic acid synthetase	COG1083, NeuA, CMP-N-acetylneuraminic acid synthetase, Cell envelope biogenesis, outer membrane, M.
**Hac1270**	413	---	---	---	---	UDP-N-acetylglucosamine 2-epimerase, NeuC1	COG0381 WecB, UDP-N-acetylglucosamine 2-epimerase, Cell wall, M.
**Hac1271**	345	---	---	---	---	N-acetylneuraminic acid synthetase, NeuB1	COG2089, SpsE, Sialic acid synthase, Cell envelope biogenesis, outer membrane, M.
**Hac1334**	157	---	---	---	---	Hac prophage I orf1 integrase	COG0582, XerC, Integrase, DNA replication, recombination, and repair, L. cd00801, INT_P4, Bacteriophage P4 integrase.
**Hac1335**	532	---	---	---	---	Hac prophage I orf2 helicase	COG4889, Predicted helicase, General function prediction only, R.
**Hac1336**	1080	---	---	---	---	Hac prophage I orf3 helicase	COG4889, Predicted helicase, General function prediction only, R.
**Hac1337**	68	---	---	---	---	Hac prophage I orf4 putative tautomerase	COG1942, Uncharacterized protein, 4-oxalocrotonate tautomerase homolog, General function prediction only, R.

**Hac1339**	69	---	---	---	---	Hac prophage I orf5 hypothetical protein	COG5055, RAD52, DNA replication, recombination and repair, L.
**Hac1340**	88	---	---	---	---	Hac prophage I orf6 hypothetical protein	n.d.
**Hac1341**	176	---	---	---	---	Hac prophage I orf7 conserved hypothetical protein[p]	pfam06067, DUF932, Family of Proteobacteria proteins with domain of unknown function.
**Hac1342**	128	---	---	---	---	Hac prophage I orf8 hypothetical protein	n.d.
**Hac1343**	159	---	---	---	---	Hac prophage I orf9 hypothetical protein	n.d.
**Hac1385**	65	---	---	---	---	hypothetical protein	n.d.
**Hac1413**	67	---	---	---	---	hypothetical protein	n.d.
**Hac1467**	94	---	---	---	---	hypothetical protein[HGT]	n.d.
**Hac1602**	46	---	---	----	---	Hac prophage II orf1 hypothetical protein	n.d.
**Hac1604**	82	---	---	---	---	Hac prophage II orf2 hypothetical protein	n.d.
**Hac1606**	378		---	---	---	Hac prophage II orf3 bacteriophage-related integrase	COG4974, XerD, Site-specific recombinase XerD, DNA replication, recombination, and repair, L.<br>cd01189, phage and phage-related integrases.
**Hac1607**	58	---	---	---	---	Hac prophage II orf4 hypothetical protein	n.d.
**Hac1608**	109	---	---	---	---	Hac prophage II orf5 hypothetical protein	n.d.
**Hac1609**	315	---	---	---	---	Hac prophage II orf6 hypothetical protein	n.d.
**Hac1610**	89	---	---	---	---	Hac prophage II orf7 hypothetical protein	n.d.

**Hac1611**	383	---	---	---	---	Hac prophage II orf8<br>CUP0976-like hypothetical protein	COG0467 RecA-superfamily ATPases implicated in signal transduction, Signal transduction mechanisms, T.<br>COG0468, RecA, RecA/RadA recombinase, DNA replication, recombination, and repair, L.
**Hac1612**	523	---	---	---	---	Hac prophage II orf9<br>DNA primase DnaG	COG0358, DnaG, DNA primase (bacterial type), DNA replication, recombination, and repair], Replication, L.
**Hac1614**	530	----	---	---	---	Hac prophage II orf10<br>hypothetical protein	n.d.
**Hac1615**	1739	**[HP1116, C-terminal]**	957	**[jhp1044, C-terminal]**	1154	Hac prophage II orf11<br>conserved hypothetical mosaic CUP0956/HP1116/jhp1044-like protein	n.d.
**Hac1616**	416	---	---	---	---	Hac prophage II orf12<br>conserved hypothetical protein	n.d.
**Hac1617**	190	---	---	---	---	Hac prophage II orf13<br>hypothetical protein	n.d.
**Hac1618**	131	---	---	---	---	Hac prophage II orf14<br>hypothetical protein	n.d.
**Hac1619**	195	---	---	---	---	Hac prophage II orf15<br>hypothetical protein	n.d.
**Hac1620**	182	---	---	---	---	Hac prophage II orf16<br>conserved hypothetical protein	n.d.
**Hac1621**	380	---	---	---	---	Hac prophage II orf17<br>conserved hypothetical protein	n.d.
**Hac1622**	119	---	---	---	---	Hac prophage II orf18<br>hypothetical protein	n.d.
**Hac1623**	143	---	---	---	---	Hac prophage II orf19<br>hypothetical protein	n.d.
**Hac1624**	600	---	---	---	---	Hac prophage II orf20	n.d.

						conserved hypothetical mosaic CUP1551/CUP0957-like protein	
**Hac1625**	517	---	---	---	---	Hac prophage II orf21 phage-related CUP0950-like protein	COG5410, Uncharacterized protein conserved in bacteria, Function unknown, S.
**Hac1626**	59	---	---	---	---	Hac prophage II orf22 hypothetical protein	n.d.
**Hac1627**	67	---	---	---	---	Hac prophage II orf23 hypothetical protein	n.d.
**Hac1628**	111	---	---	---	----	Hac prophage II orf24 hypothetical protein	n.d.
**Hac1629**	73	---	---	---	---	Hac prophage II orf25 hypothetical protein	n.d.
**Hac1630**	181	---	---	---	----	Hac prophage II orf26 hypothetical protein	n.d.
**Hac1631**	261	---	---	---	---	Hac prophage II orf27 hypothetical protein	n.d.
**Hac1632**	180	---	---	---	---	Hac prophage II orf28	n.d.
**Hac1633**	95	---	---	---	---	Hac prophage II orf29	n.d.
**Hac1634**	294	---	---	---	---	Hac prophage II orf30 hypothetical protein	n.d.
**Hac1635**	281	---	---	---	---	Hac prophage II orf31 conserved hypothetical protein	n.d.
**Hac1636**	276	---	---	---	---	Hac prophage II orf32 hypothetical protein	n.d.
**Hac1662**	82	---	---	---	---	hypothetical protein	n.d.
**Hac1699**	76	---	---	---	---	hypothetical protein	n.d.
**Hac1707**	185	---	---	**[jhp0949]**<br>**[jhp0948]**	420<br>92	hypothetical protein with type IV secretory domain	COG3846, Type IV secretory pathway TrbL components, Intracellular trafficking, U.
**Hac1759**	74	---	---	---	---	hypothetical protein	n.d.
**Hac1762**<br>**Hac1763**	258<br>147	**[HP1017]**	519	**[jhp0406]**	475	amino acid permease RocE’	COG0833, LysP, Amino acid transporters, Amino acid transport

**Hac1764**	44						and metabolism, E.
**Hac1782**	298	---	---	---	---	putative type II DNA methylase protein$^{R/M}$	n.d.

The cluster of orthologous groups (COG) category and respective gene names is listed even if not detectable in all fragmented open reading frames. ‘ duplicated in *H. acinonychis* Sheeba, HGT, horizontal gene transfer, R/M restriction/modification system, n.d. no putative conserved domains have been detected, p, plasmid, pg paralogous gene, pp, prophage, omp, outer membrane protein. Prophage I and II are regarded as distinct *H. acinonychis* Sheeba specific epsilon-proteobacterial prophages. Therefore the phage encoded proteins were categorized as unique genes, even if there is homoloy to orthologous phage proteins in *C. upsaliensis* RM3195 or homology to DNA-modifying proteins in the epsilon-proteobacterial genome pool. Unique genes are often clustered which might be reminiscent of the integration process via horizontal gene transfer.
