# Supplementary material for: Who Ate Whom? Adaptive Helicobacter Genomic Changes That Accompanied a Host Jump from Early Humans to Large Felines
Source: PLoS Genet. 2006 Jul 28;2(7):e120. doi: 10.1371/journal.pgen.0020120 (PMC1523251; doi:10.1371/journal.pgen.0020120)
Supplement: Table S5 — (56 KB DOC) [file pgen.0020120.st005.doc]

**Table S5. Sources of *H.* *acinonychis* and *H. pylori***.

| ***H. acinonychis* complex** | | | |
| --- | --- | --- | --- |
| **Strain** | **Origin** | Host | **Reference** |
| Sheeba | Russian circus | lion | Dailidiene *et al.*, 2004 (*S2*) |
| Bombay A | Russian circus | tiger | Cattoli *et al*., 2000 (*S3*) |
| Bombay B | Russian circus | tiger | Cattoli *et al*., 2000 (*S3*) |
| India | Russian circus | tiger | Cattoli *et al*., 2000 (*S3*) |
| t1 | German zoo | tiger | Schroder *et al.*, 1998 (*S4*) |
| t2 | German zoo | tiger | Schroder *et al.*, 1998 (*S4*) |
| HA5141 | USA zoo | cheetah | Eaton *et al.*, 1993 (*S5*) |

| *H. pylori* complex | | | |
| --- | --- | --- | --- |
| **Strain** | **Geographic origin** | ***H. pylori* (sub)population** | **Reference** |
| SU2 | Sudan | hpEurope | Falush *et al.*, 2003 (*S6*) |
| fin9625 | Finland | hpEurope | unpublished |
| leb3438 | Lebanon | hpEurope | unpublished |
| L128 | India | hpAsia2 | Gressmann *et al*., 2005 (*7*) |
| L15 | India | hpAsia2 | Gressmann *et al*., 2005 (*S7*) |
| L67 | India | hpAsia2 | Gressmann *et al*., 2005 (*S7*) |
| cc42c | South Africa | hspSAfrica | Falush *et al.*, 2003 (*S6*) |
| C164 | South Africa | hspSAfrica | Falush *et al.*, 2003 (*S6*) |
| D3a | Senegal | hspWAfrica | Falush *et al.*, 2003 (*S6*) |
| LSU2003-1 | USA | hspWAfrica | Falush *et al.*, 2003 (*S6*) |
| 129.0 | South Africa | hpAfrica2 | Falush *et al.*, 2003 (*S6*) |
| 162.0 | South Africa | hpAfrica2 | Falush *et al.*, 2003 (*S6*) |
| SA36C | South Africa | hpAfrica2 | unpublished |
| N2 | Korea | hspEAsia | Falush *et al.*, 2003 (*S6*) |
| RE8030 | Singapore | hspEAsia | Falush *et al.*, 2003 (*S6*) |
| M49 | New Zealand | hspMaori | Falush *et al.*, 2003 (*S6*) |
| inma50 | New Zealand | hspMaori | Falush *et al.*, 2003 (*S6*) |
| HUI1692 | Colombia | hspAmerind | Falush *et al.*, 2003 (*S6*) |
| HUI1764 | Colombia | hspAmerind | Falush *et al.*, 2003 (*S6*) |
| inma10 | Canada | hspAmerind | Falush *et al.*, 2003 (*S6*) |
| inma14 | Canada | hspAmerind | Falush *et al.*, 2003 (*S6*) |
