# Supplementary material for: Who Ate Whom? Adaptive Helicobacter Genomic Changes That Accompanied a Host Jump from Early Humans to Large Felines
Source: PLoS Genet. 2006 Jul 28;2(7):e120. doi: 10.1371/journal.pgen.0020120 (PMC1523251; doi:10.1371/journal.pgen.0020120)
Supplement: Table S6 — (43 KB PDF) [file pgen.0020120.st006.pdf]

Table S6. Primer pairs for microarrays.

Gene	forward primer	reverse primer
Hac0028	ATGTTTTTAATTCTGTCTTGT	TTAAGAGGTTAAAAACAACC
Hac0101	TTGGACAATGAAATCACAAGC	TCAAGAGTGTTCCCTTAAGC
Hac0111	AAAGTTGCCCCACAAACG	AAATCAACAGCAAGCTCAA
Hac0118	AAACCATTCCGGCTAAACAT	CTTTTGGGATTAGTCGTT
Hac0128	TCTCGCATTTAGAAAATCCG	GCCACCAATCTCCCAAAA
Hac0130	ATGCTTTGCCTTTTTGAGGAC	CTACTTAAAAACGCCATTTTT
Hac0162	GGATCGTTTGGGTCGTCTAA	AAAATAGAAACGCTCGTTCCTG
Hac0208	GCTGTTATCGCCTATTGCA	GACATCTCGTTGTTCCCAAAA
Hac0283	GAAGTAGGGAAGCCGGTAATG	CTGGTGGTTCGCATTTAGG
Hac0284	ATGCCTTATAGACTAGAAAAAG	GCCATTTAAGATTTCCTCATATC
Hac0322	CGATTGCGTATTTGATCC	ATGCATGCAAAAATCCGAG
Hac0360	CTGATGAATTGATCAATGGC	AATTGACTGGCACTGCATTG
Hac0361	TAGCTTGTGAAGAAAGCC	CTTTTGCAAATCCTGGGAA
Hac0425	ATGAATATCCCTAACTTACCT	TCAATGTTTTGATAACAATCGT
Hac0447	CAACGGTGAGTTTTCCTGTG	ACAGAGCTTTGGGCAGAAGT
Hac0448	TGTAAGCCCATTCATGCCTA	TATGCAGGAGAAGTTGCCAG
Hac0498	CATGTTGAAAACTTGGGAGC	TACCGATTTGTCTGCCATAT
Hac0499	CAAATCGGTAAATGAACCGA	GCATCATCCGTGTCTAATGG
Hac0567	GGCTTGTTTGGTTTCTCG	GAAAGTGAGGGCGGTAAGC
Hac0569	CTCTTTCACCCTCGCTGC	CAACTCTATGCCATGCTGGA
Hac0572	AAACACCCACACCACATTGA	TTTCGCAAACCCATACACTTC
Hac0591	AACCTCAAAAATTGGTTG	TTCCTTACCAAATTGCTTGG
Hac0620	GGGACAGCTGAAAAGTTTGC	ATTAAGAACCCTGAATGGTA
Hac0621	GGCGGTAAGGTTTATGCT	GAGCTAGGGTTCATATCG
Hac0622	ATGAAAAAATTTCAACATTACAAGC	CTAGAAAGGAGCGGTTATGC
Hac0623	TTGTATAAACACCTGCTTAACG	CAAAGAACGCGAATTTTAAAGG
Hac0709	CTCTTTGGTTGTTGTTGTGC	TTAGAAAATGCAAAAAATTTTTGAA
Hac0710	GAAACAGAGAACGCCCAGTT	TTGTTGTCGAACTTTTACCACCT
Hac0711	AACTCAAATGATAGCGCA	AAGGGAGGGAGTCCGTATAGA
Hac0712	AAAGCACCAACTGGCAGTG	ACTCCAAATTTGCCAATCGT
Hac0757	GTGGAATTTGAACAGATTAGAA	TTAGGGCGCTTTAGTTAGGG
Hac0762	GTGTGGTGTCCTAAATACAGA	TTAGTGTAGAGCTTTTGTTTGT
Hac0765	GTTGGAGAAAATATGAGCAAC	CAATATTGCTTGTTATTGCCC
Hac0766	CAATTCCAAATCGGCAATT	TTTACTGCATGTGTTGGTC
Hac0776	CAGCGGTAATTAAAGTCCCAA	TTGGGATAAAAGTCGCCCT
Hac0837	TTCAACTTTTATGCGGGT	AATGCCCATGTCATAGGC
Hac0852	TGCAGGAGCTAGAATAAGCA	TTCTCCTTATTTGCTTAGTTGA
Hac0853	GTGAAGGCATTCTGTGGAAT	AGCGAGCATCCTATCGAGAG
Hac0887	CACAAGTTACAGAGATTGATCC	ACCCACGATGATGAGCTACT
Hac0889	AAAATTCCCTTATGACAATGCC	TCTTGGTGATAAAGCGCC
Hac0890	TTGCCCCAAGACTTTCTC	CCTTTACACAAATCAGCTTGGA
Hac0891	GTGTTGCAAGAGATTAGAGAA	TTGCATATAAGATTGAATCCAC
Hac0921	GTATAAACACCTGCTTAACGA	CAAAGAACGCGAATTTTAAAGG
Hac0922	ATGTCTAGCGATTTCAAAAAC	CTAGAAAGGAGCGGTTATGC
Hac0935	TTATCGCAACTTTATTCCCT	AAACATCAAGGCTCTCCCTG
Hac0965	AACCGCTTTAGATTCTGC	TTTTGTATCCACTCCACATCGTA
Hac0966	CGATATGGGGTGTACTCTCG	TGAATTGGACAAAGACGCAT
Hac1016	TACGCGCCAAATGAGATAAC	TCTGCGCCCTAATTGTTTG
Hac1017	GATATTATACAAGATTATAGTGTTG	CTTTCAATTCATTTTCCATTAGTT
Hac1112	GACTAATATGACTAAACTTTTAG	CCCAAAGTGGCGTTGGTTG
Hac1213	TCATTATTTGCTGGAGCCG	TGAATCCTTGCACACTCTCG
Hac1214	GGCGTATGAATACGCATGG	GTTATAAAGACTGCAACACTC
Hac1267	GCCGGCAACGGTCCTAGC	GCCTTTATGATGGATTTGAGC
Hac1269	TTGTCTATATCTAGGGCATGA	CCTACATTCCTGCCAGAAGC
Hac1270	CGTAGGCGTGTCAAACAATT	AACTCTTCCATTTTTCGGAGG
Hac1271	TTCATCGCAAATGTGTTCG	TGGTATCAACCACACGCCT
Hac1334	GATGAGAGGATGAGCCCAAG	TGCTTTAGCTCTCCAAGCG
Hac1335	CGCTACGAACGCTTACTTCA	GGCGACTATCTGAGCAGAGAA
Hac1336	TTGCTTGTCCTAGCTGGTGA	TATAGTCTGCGATGAAGCGC
Hac1339	TTGCACTACAAGACTATTGTTA	ATAAAGCGAATTACCAAACTGAT
Hac1340	TCACTTGCGCTTTGGTCTT	TGCTTAGACTTGGCCATGTC
Hac1341	AGCTGGCGAAGCTTATCTTG	GCAACAACGAGCTAAAACGAC
Hac1342	GATTGGTCGCTTTTCCAGTT	GGTGATAACGGTCAACAGTCAA
Hac1343	TTCTTATAACTTGCTTGCCTC	AATAATCCTAAAGGTGGACA
Hac1413	ATATTCCTCAAGCGAAGTGC	GCTAAGGGAAAGTGCCACAG
Hac1466	TTGATTAGAACCCCTAAACAT	CAAAACTCTTTATTGCCATAAGC

Hac1467	ATGGCTACAAGCGTTAATTTAG	GGAATAATGCCTTTTGCTCG
Hac1487	GTGTCCTAAATACAGACGCA	GTGTAGAGCTTTTGTTTGTAG
Hac1513	GCATCTTTTCATCACCTTCC	GGAGAGAGACTTGCACCCAT
Hac1514	ATGACTTACAAAGAATTAGGTAA	CATTTAAACATTCGCTCTCTG
Hac1606	AATGCCTGCAAATGAAGCTT	CTCTGTCTTGTTGCGGGATA
Hac1607	TGAAAGCGGAGAAAACGG	CCCTTTCTTTGCAACATGA
Hac1608	ACTGCATCTGCAACGACC	CATTGGAGATCCTTTGTGTGTT
Hac1609	CCATGCAATACGGTTACGTG	CCTACCAGATGCAATAGCCA
Hac1610	CATGGAAAAAATCCAAACGAG	CTTTGGTTTTTAGCTTCTAAAAC
Hac1611	TTAGACTACCCCGAACGCAT	CCGGTCTGCTTGTTCTTCA
Hac1612	GTTTCATGGTGAGCAGGGA	GGAATTCAAAGGGAGAGGCT
Hac1614	AGCGCGCCTTAGAACAAAT	CAGCAACAAACGGGTGAGT
Hac1617	GCTTAACGGGGGTTTTCA	TTCAAAGGCTTCCACCTC
Hac1618	GTGTTTAATGCAAGCGGG	TGCATTTCCCTTTCACAA
Hac1619	GATCAAAGTCAGGGAACGGT	TGCGTCAGGTTGTCTTTAGC
Hac1620	AGAAATCGAATTGGAAACCT	ATCTTGTCATCACGCTGTCG
Hac1621	TAGGGGTAGAAATCGGCAAA	CATCCACGCTCTGATACCTG
Hac1622	ATGAAACAAAAAGTCCATAGCG	ATATTCCACTTCCACCACGC
Hac1623	ACGATCCGCATTATTTCAGG	CAAGCGCTGCTTCTTTGTT
Hac1624	AATTACCGCCAGACGTTTTG	TTCTGCGCGCTTCTGTATT
Hac1625	AGTGGGAACGCTACGAAAAC	ATCAAATCCTCCATGTCGGT
Hac1626	ATGAAAAAAAATCGTATCATCAG	TCACAACCTGCCATTCAAAC
Hac1627	ATGTGGGATGAAAAAATTTTGAAA	CATGCCTTGAGCATGGATG
Hac1628	GTTTTGAGTTATGAGATGGC	TACTTAAGGTCTCAGCGCCC
Hac1630	GAAGATTTGAAGCCTTTAGTG	CAAACCTTATCTATGATTTTGAA
Hac1632	CCAAGCGTTGGATAACCA	AGCGCAAACTCTTCTTTG
Hac1634	GAGGAACGAACTCAACGAGC	TCCAGTTTGCCTTCGCTAAT
Hac1635	AACAATTGAAGCTGGCGC	TAAGATCGATTTTCCCGCC
Hac1636	GGTGAGGGAAGCGTATGAAG	AGTTGGGATGCGCTATCG
Hac1707	AGGAAGATAACACAAACTAGCG	AAAGCGGATTAAACTCATGG
Hac1762	CTGATAGCCCCATTGGTTT	TTGTTATTGTGACAGCGAT
Hac1763	GCCCTTCTCTAGCCCTTG	CGTGTATCCTACCACAGGGA
Hac1782	CGCTTTTTACACCCACCC	CCCCAAAACTTGCGTGTT
