# Supplementary material for: Who Ate Whom? Adaptive Helicobacter Genomic Changes That Accompanied a Host Jump from Early Humans to Large Felines
Source: PLoS Genet. 2006 Jul 28;2(7):e120. doi: 10.1371/journal.pgen.0020120 (PMC1523251; doi:10.1371/journal.pgen.0020120)
Supplement: Table S7 — (86 KB DOC) [file pgen.0020120.st007.doc]

**Table S7. PCR-primer pairs.**

| **Gene** | **Primer** | **Sequence (5’-3’)** | **Product [bp]** |
| --- | --- | --- | --- |
| *omp1*  (Hac0035-38) | omp1_1 F | ATACCGCCATAAACCCCCACAGA | 1276 |
|  | omp1_1 R | CTTCGCCCGCGTATCAGAC |  |
| *omp1* | omp1_2 F | CTTTACCGCCATCGCTTGTTTCT | 1100 |
|  | omp1_2 R | TTTTTCACGCCGGTTTTTGTCAT |  |
| *hypothetical protein* 1  (Hac0690-93) | hp_1 F | GGCTTAGGGGCTTCAATAGGAG | 1056 |
|  | hp_1 R | ACAACAGCGACAATCACCAAACT |  |
| *hp* | hp_2 F | AACCCGTCAAATCCCATCAA | 873 |
|  | hp_2 R | CCACAGCCACTATCCCATCTCTA |  |
| *hp* | hp_3 F | ACGCCGATCTGAGTTCTTCTGA | 1063 |
|  | hp_3 R | GCTGCCTTTCTTGATTGTTGAGTT |  |
| *hypothetical protein* 2  (Hac0731-2) | chp_1 F | GACGATTGGCTTTGCACGACATT | 823 |
|  | chp_1 R | AACGCCGAACCACCAAGAGAAGA |  |
| *chp* | chp_2 F | ATATTCGCTCCTGGCTTTCACTTC | 1312 |
|  | chp_2 R | AGCGGGTTACTCAATCTTACAGGA |  |
| *chp* | chp_3 F | ATACACTTCGCGCTCTTCCTCTTT | 1111 |
|  | chp_3 R | TACGCGTGCGATAATGGTTTT |  |
| *omp12*  (Hac0942-6) | omp12_1 F | TACCCGAACCTCTTAACCCACTGA | 1155 |
|  | omp12_1 R | GCACGACTCCCAATCCCAATG |  |
| *omp12* | omp12_2 F | GTGCCAGCGAATGCGATACC | 1475 |
|  | omp12_2 R | ACCGGCGAATTGAAAAACTTGAAC |  |
| *omp14*  (Hac1007-10) | omp14_1 F | AGCCCAAACGCCATCAATAAGTGC | 1037 |
|  | omp14_1 R | AAGCGTTCAAATCAGGCGTGTCAT |  |
| *omp14* | omp14_2 F | AAGGAAGGCGGCGCTCATAGG | 693 |
|  | omp14_2 R | GAAAATCCGCCGCATCCACTT |  |
| *homB*  (Hac1244-7) | homB_1 F | AAAACAAAGCCAAAATAACACCAA | 846 |
|  | homB_1 R | AAGCCGGGGAATCAAACACT |  |
| *homB* | homB_2 F | CGACGGCTTTATTCACCACAA | 949 |
|  | homB_2 R | CCCTTCCGCCTTCACATTCT |  |
| *homB* | homB_3 F | CTTTCAATAATGGGCGTGTCG | 1263 |
|  | homB_3 R | AAAATGATAAAATGCGGAATGGAT |  |
| *omp20*  (Hac1278-81) | omp20_1 F | CCTTATTTTTGGCGGGATTCACC | 977 |
|  | omp20_1 R | GCCGCGCTCAAAAAGTCGTAAC |  |
| *omp20* | omp20_2 F | CTATGAGCGCCGCCTTCCTTGAT | 1284 |
|  | omp20_2 R | TGCACCCCAGCCATTCTTTTTACT |  |
| *omp28*  (Hac1386-89) | omp28_1 F | ACGGCTTTATTCTCACGCATTCAT | 1414 |
|  | omp28_1 R | TTCCCCCAAGTGGTATTTTCATCT |  |
| *omp28* | omp28_2 F | GCAATCGCAAGCAGAAA | 1183 |
|  | omp28_2 R | CATCAAATTGGGCGAGAG |  |
| *omp30*  (Hac1489-91) | omp30_1 F | TAGGGGCTTTATGGCGTTAGG | 1114 |
|  | omp30_1 R | TTTGGCGAATTGCTTGTTGTTG |  |
| *omp30* | omp30_2 F | ATCCGAATACAACACCCAGAC | 1222 |
|  | omp30_2 R | AGCATTTTAGCGACTTTACACAA |  |
| *vacA*  (Hac1253-66) | vacA_for1 | GCTTTGATGGACACCCCACAAGG | 10281 |
|  | vacA_rev2 | GCAAGCTTCCACGCMAATCGCATGACTTC |  |
|  | vac-for4-Ha | CAAACACACCATAAACAC |  |
|  | vac-rev4-Ha | GTGTTTATGGTGTGTTTG |  |
|  | vac-for5-Ha | GGAGAGGCTTTGATTCTT |  |
|  | vac-rev5-Ha | AAGAATCAAAGCCTCTCC |  |
|  | vac-for6-Ha | GTATGAAAAACGCCGTAG |  |
|  | vac-rev6-Ha | CTACGGCGTTTTTCATAC |  |
|  | vacA_rev1 | GCTTGAATGCGCCAAACTTTATC |  |
|  | vacA_rev2 | GCGTCTTTCATATCCACTTCAAG |  |
|  | vacA_for1 | GYGTGGGTTCTGGAGCGGG |  |
|  | vacA_for2 | CGCTTCCAATTTAGGAATGAGGTA |  |
